# Cuticular hydrocarbon profiles in plump bush crickets vary according to species, sex and mating status

**DOI:** 10.1038/s41598-025-17544-7

**Published:** 2025-09-26

**Authors:** Hasan Sevgili, Emine Bağdatlı, Elif Açıkel

**Affiliations:** 1 Department of Molecular Biology and Genetics, Faculty of Art and Science, Ordu University, Ordu, Turkey; 2 Department of Chemistry, Faculty of Art and Science, Ordu University, Ordu, Turkey

**Keywords:** Cuticular hydrocarbons, Chemical ecology, Sex, Mating status, *Isophya*, Orthoptera, Chemical ecology, Evolution

## Abstract

Cuticular hydrocarbons (CHCs) serve critical roles in insect communication and desiccation resistance and are increasingly recognized as valuable taxonomic characters. This study investigates inter- and intraspecific variation in CHC profiles across ten *Isophya* species representing three distinct species groups (*zernovi*, *rectipennis*, and *staneki*), focusing on how these profiles vary by species identity, sex, and mating status. A total of 829 individuals (411 females, 418 males) were sampled and analyzed via GC–MS to quantify CHC composition. Multivariate analyses revealed strong effects of species and sex, as well as significant species × sex × mating status interactions. In both males and females, species in the *zernovi* group displayed tightly clustered CHC profiles, whereas members of the *rectipennis* group exhibited broader within-group dispersion, with *I. rectipennis* forming a distinct cluster. *I. staneki* was clearly differentiated from all other taxa. CHCs were categorized into six structural classes, with n-alkanes being the most dominant across all taxa. Linear mixed-effects models confirmed that CHC class composition was significantly affected by sex and mating status, particularly for alkenes and methyl-branched alkanes. Notably, nonvirgin individuals showed greater CHC variability, suggesting reproductive condition influences chemical expression. While the study remains descriptive, these findings highlight the potential utility of CHCs in taxonomic resolution, sexual communication, and ecological adaptation in *Isophya*. The integrative use of CHC data, in combination with morphological and acoustic traits, provides a promising framework for understanding species boundaries and evolutionary divergence in Orthoptera.

**Supplementary Information:**

The online version contains supplementary material available at 10.1038/s41598-025-17544-7.

## Introduction

Habitat differentiation and niche partitioning significantly contribute to the evolutionary divergence of chemical signals and speciation in insects. The diversification of these signals across populations and species is particularly pronounced in environments with varying climatic or habitat conditions^1,2^. Insects have developed intricate cuticular chemistry, which is primarily composed of hydrophobic hydrocarbons of different chain lengths (cuticular hydrocarbons, or CHCs), enabling them to thrive in terrestrial environments^3^. Insects communicate via chemical signals, which can include CHCs or volatile compounds^4^. Cuticular hydrocarbons are perceived through direct contact or short-range proximity^4–6^. Male *Chorthippus* grasshoppers use CHCs to recognize potential mates and touch the body and antennae of females with their antennae before mating^7^.

In addition to their role in communication, insect CHCs provide essential protection against microbial infections, environmental stress, and desiccation by covering the entire body surface with multiple layers of compounds^3,8^. These compounds exhibit intra- and interspecific variation and sexual dimorphism, allowing them to be modulated on the basis of environmental conditions^9–13^.

CHCs, comprising a complex blend of unsaturated hydrocarbons, methyl-branched alkanes, and n-alkanes secreted from arthropod cuticles, also play a vital role in biochemical taxonomy, particularly among social insects^11,14–16^. Researchers have documented them across various other insect groups, such as grasshoppers﻿﻿ ﻿﻿*Chorthippus*﻿﻿﻿﻿﻿﻿﻿ ﻿﻿sp.,^17–20^, field crickets *Gryllus* spp.,^21^, ants^22–24^, various Hymenoptera taxa^25^, and fruit flies﻿﻿ ﻿﻿﻿﻿﻿﻿﻿﻿﻿﻿﻿﻿﻿﻿*Drosophil﻿a*﻿﻿﻿﻿﻿﻿﻿﻿ ﻿﻿sp.﻿^14,26–29^. Despite their widespread occurrence, CHC profiles are challenging for evolutionary biologists because of the striking diversity in composition, even among closely related species^12,16,21^. This complexity in CHC profiles is a fascinating area of study, demonstrating the intricate roles of CHCs in ecological adaptation and species recognition, underscoring their evolutionary importance across insect taxa.

The reasons for the diversity of CHC profiles in insects remain largely unknown, and the understanding of the selection pressures shaping these profiles is limited. Given the multifunctional nature of CHCs, they may not only mediate interactions within species (e.g., social insects) but also contribute to a species’ ecological niche, encompassing both abiotic factors and biotic interactions. As a result, CHCs may play a role in niche partitioning, especially among closely related species. For example, Schwander et al.^30^ indicated that changes in CHC profiles can drive speciation, with directional selection on the basis of mate choice influencing divergence processes. Additionally, Rajpurohit et al.^28^ highlighted that CHCs play a crucial role in desiccation resistance, which may impose selection pressures on these profiles. Furthermore, the interaction between insect pheromones and plant defenses, as discussed by Bittner et al.^31^, may further complicate our understanding of CHC diversity dynamics.

Studies on various insect species, including the Argentine ant^32^, the Navel Orangeworm^33^, and the Western honey bee^34^, highlight the adaptability and variation of CHC profiles in response to environmental conditions, genetic factors, and social roles. Despite this variability, CHCs also maintain conserved functions, such as colony recognition and communication, often balancing the need for environmental adaptation with maintaining uniform chemical signatures across populations. These findings underscore the multifaceted roles of CHCs and their potential as biomarkers for ecological and pest management applications, offering promising solutions for practical challenges in the field.

In line with the evolutionary importance of CHCs, the species belonging to the genus *Isophya* (Orthoptera: Tettigoniidae) provide a valuable case study to investigate the evolutionary and ecological reasons for the differentiation of these hydrocarbons among species, especially in regions where researchers have observed high levels of endemism. Under these conditions, a species is unique to a defined geographic location, such as Anatolia or the Balkans. Orthopterists have reported 90 species of the genus *Isophya* worldwide^35^, with 44 found in Anatolia, where an average of 77% are local endemics^12,36,37^. Owing to their short wings, *Isophya* species have limited mobility, and their adult lifespan, which lasts for several months, is confined to a small area^38^. The overall body morphology of *Isophya* species is similar, and the taxonomic characteristics used for identification are limited, making distinguishing between species with apparent morphological differences challenging. However, in males, the pronotal structure, wings, sound-producing organs, and cerci present relatively distinct features across many species.

In contrast, females possess fewer taxonomically functional structures compared to males. Given these taxonomic difficulties, male calling song characteristics—both temporal and structural—and molecular studies have been frequently employed to differentiate between *Isophya* species^36,39–45^. However, complete concordance between morphology, bioacoustic, and molecular findings is rare among species or species groups^42,43^.

The differentiation and specialization of CHCs among insect species are key components of species-specific communication and behavioral isolation^15,16,18,46^. CHC profiles vary significantly across species, comprising a unique blend of hydrocarbons that differ in chain length, saturation, and methyl branching^14,18,47,48^. This chemical diversity suggests that CHC composition evolves through regulatory and genetic modifications, potentially driven by mutations in biosynthetic pathway genes or gene duplications^8,18^. Sexual dimorphism in CHC profiles, though not always pronounced, significantly influences sexual selection, mediating differential courtship responses and affecting mating outcomes between sexes^11,21,27,49^. It mediates differential courtship responses and affects mating outcomes between sexes.

The objectives of this study are to focus on several key aspects of CHC variation in *Isophya* species. First, it aims to investigate how CHC profiles differ between species within two selected *Isophya* species groups, providing insights into interspecific variation. Second, it analyzes CHC differences at the species group level to uncover differentiation patterns or similarities within and between these groups. Additionally, this study explored sex-based variation in CHC profiles, particularly potential sexual dimorphism in chemical composition. Finally, we explore how mating status is associated with variation in the composition and abundance of CHCs. While our data are descriptive, the observed patterns may inform future studies on the potential roles of these hydrocarbons in reproductive behavior and chemical communication in *Isophya*. Overall, this study provides a comparative framework to investigate how CHCs vary across species, sex, and mating status—factors potentially linked to species recognition and sexual selection.

## Materials and methods

### The plump bush crickets

Species of the *Isophya* genus are plump, short-winged bush crickets (see Supplementary Fig. S1) that inhabit high-altitude grasslands with dense green herbaceous vegetation. *Isophya* species often inhabit areas mixed with *Juniperus communis* and *Astragalus* spp., as well as forest interiors and edges, where they commonly occur on nettles and blackberries^12,36^. Owing to their restricted movement ability, individuals primarily stay within small, localized areas, making them an ideal model for studying chemical communication, reproductive behaviors, and habitat-specific adaptations.

This study was conducted on several species belonging to the *I. zernovi* species group, which is distributed in the Eastern Black Sea region and Northeast Anatolia, and the *I. rectipennis* species group, which is found in the Central and Western Black Sea regions as well as the northern areas of Central Anatolia. These species are considered distinct species groups on the basis of their morphological and molecular differences^36,43^. A photographic collage presents representative individuals of selected *Isophya* species from various sampling localities (Supp. Figure 1), highlighting morphological diversity across the *zernovi* and *rectipennis* species groups, as well as evident sexual dimorphism between males and females. Additionally, *I. staneki*, a member of the *I. straubei* group, exhibited significant morphological distinctions from the other two species groups (including differences in the fastigium, pronotum, male wing, and cercus morphology)^36^ and was included in the analyses. *I. staneki* was included to assess the positioning of the CHC profile patterns of the two main species groups.

### Field studies

**Fig. 1:**
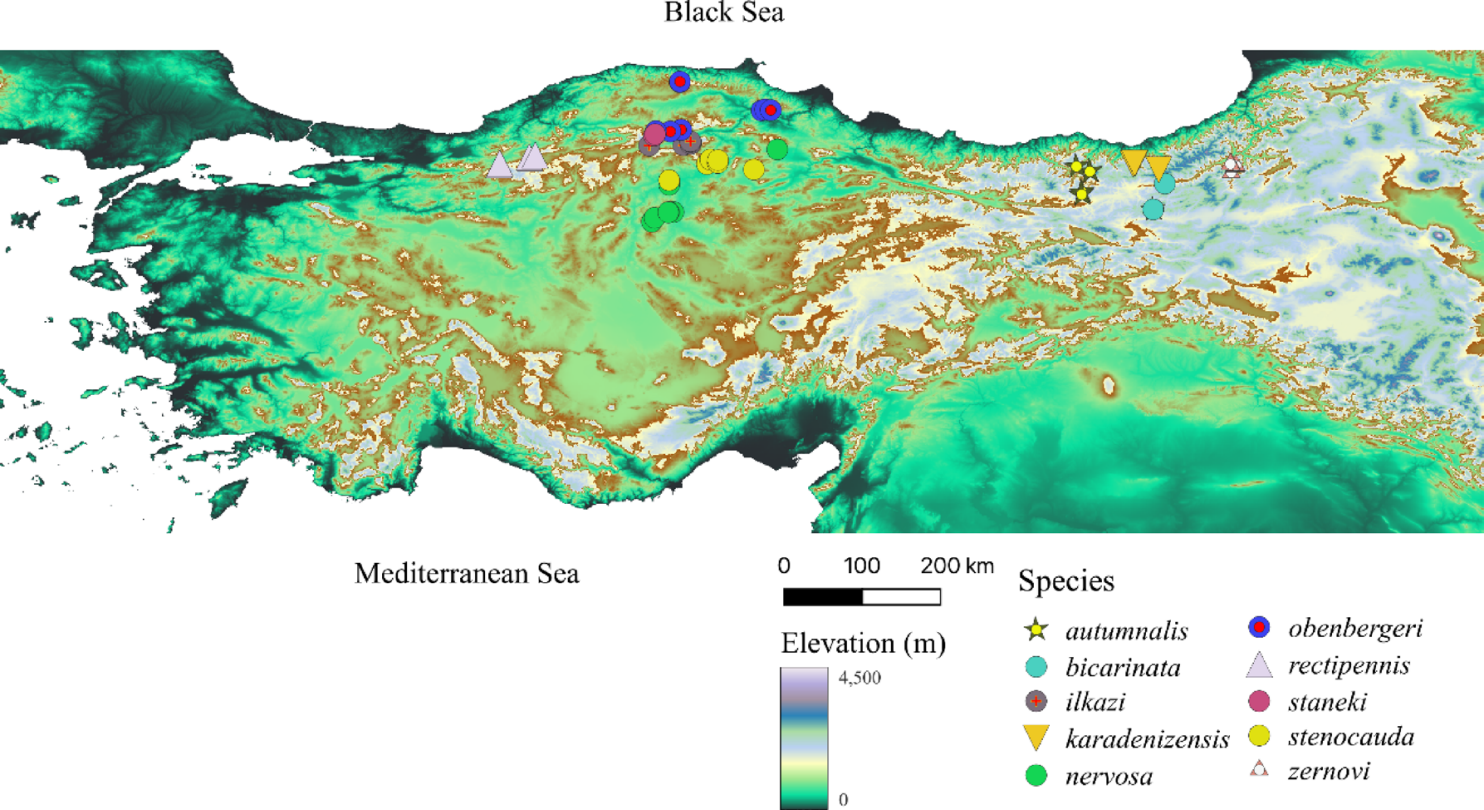
Sampling localities of *Isophya* species analyzed in this study. The figure displays the visited sites where species were identified, with sampling conducted primarily in locations with the highest population densities. The map was created in QGIS (version 3.36.3; https://qgis.org) using publicly available SRTM elevation data, and all layers, symbology, and annotations were manually edited by the authors.

We collected specimens from the *I. zernovi* group, including *I. autumnalis*, *I. karadenizensis*, *I. zernovi*, and *I. bicarinata*, from various localities in Turkey (Fig. 1). Data for *I. autumnalis* came from Gümüşhane Province, where field studies and published research had previously expanded its known distribution. The variation in CHC profiles among the subpopulations of *I. autumnalis* has been extensively studied and published, providing insights into the chemical diversity within this species^12^. Additionally, we collected *I. karadenizensis* from Trabzon, *I. zernovi* from Erzurum and Artvin, and *I. bicarinate* from Bayburt provinces. Moreover, specimens from the *I. rectipennis* species group, including *I. rectipennis* (Anatolian population of *I. rectipennis*), *I. stenocauda*, *I. nervosa*, *I. obenbergeri*, and *I. ilkazi*, were collected from regions such as Bolu, Çorum, Kastamonu, and Çankırı, with detailed fieldwork revealing additional localities and extending known altitudinal distributions for several of these species (Fig. 1). The limited number of *I. staneki* individuals studied is due to the endemic distribution of the species within a restricted area (Kastamonu Province) and its low population density.

### Maintenance of the bush-crickets

For each species, excluding *I. staneki*, 100 final-instar nymphs of both males and females were collected. The collected specimens were transferred to the laboratory under healthy conditions and were immediately placed in cages for further study. Nymphs were housed separately in species-specific cages, with males and females also kept in distinct 40 × 40 × 30 cm cages and inspected daily. Upon maturation, the nymphs were separated by sex and transferred to smaller 20 × 20 × 25 cm cages, with ten individuals allocated to each cage. We took great care to ensure the proper development of the species, cleaned the cages daily using a brush and alcohol, and maintained a hygienic environment. On the basis of the localities where the samples were collected and previous laboratory experience^12,50,51^, the bush crickets were fed local vegetation, such as blackberry thorns, nettles, and other plants and flowers. Additionally, their diet was supplemented with lettuce and carrots daily. To preserve the freshness of the collected plants, they were placed in small water-filled containers inside the cages, which functioned like small vases.

### Mating experiment

To investigate the effect of mating status (virgin vs. nonvirgin) on the cuticular hydrocarbon (CHC) profiles, we applied a standardized mating protocol for each species^50,52^. Males and females that matured under laboratory conditions were randomly assigned to either virgin or mated groups. Individuals in the virgin group were separated by sex immediately after emergence and housed in same-sex cages (10 individuals per cage) under identical feeding and environmental conditions. These individuals were kept unmated until the end of the experiment and ranged in age from 6 to 8 days. In the mated group, only virgin males and females aged 8 days were used, as individuals below this age are typically less willing to mate^52^. Mating trials were conducted in cages with a 1:1 sex ratio (e.g., 10 males and 10 females per species). To distinguish individuals, males and females were marked using small labels attached to their hind femurs. All mating pairs were given the opportunity to copulate over a period of 1 to 4 days. After mating, males and females were housed separately in individual cages under the same rearing conditions. To minimize age-related variation, individuals from both groups were preserved when all matings had been completed. The final mean age of both virgin and mated individuals was 13 days. All individuals were stored separately in Eppendorf tubes at − 80 °C until chemical analysis.

### CHC extraction and analysis

To establish the CHC profiles of the samples, we utilized methodologies previously employed in Orthoptera research^2,12,17,27,53–55^. CHC data were collected via a Shimadzu GCMS QP2010 Ultra gas chromatograph‒mass spectrometer (GC‒MS). The samples frozen at − 80 °C were carefully placed in a desiccator for 45 min to reach room temperature and eliminate excess moisture. Each sample was placed entirely in a separate conical glass centrifuge tube, and 4 ml of hexane containing 10 ppm pentadecane (the internal standard) was added. After allowing the solution to sit in hexane for 30 s and vortex for 5 min, the upper layer was extracted via disposable glass pipettes and transferred to the GC/MS for analysis. For the CHC profiles of the bush-crickets, 1 µl of each sample was injected for split-mode (10:1) analysis via a Stabilwax column (Restek/Rtx-5) with a diameter of 30 m and 0.25 mm under helium gas. The analysis commenced at 150 °C for 1 min, followed by a temperature ramp to 250 °C at 15 °C per minute. The temperature was then increased to 320 °C at 3 °C per minute and held for 5 min. The extract was separated based on temperature differences within the column. Component transfer to the GC/MS instrument was performed at 250 °C. Hexane was added daily for analysis to avoid contamination. The peak numbers were coded according to retention times for data analysis. We determined the identities of the primary hydrocarbons by consulting GC/MS libraries (NIST-17 and Willey libraries) and other published sources. Fragmentation profiles were acquired and analyzed via MS and MSD ChemStation software.

The dataset we compiled is comprehensive, containing 829 individuals, 411 identified as female and 418 identified as male, belonging to ten species. With respect to the species group distribution, the *rectipennis* species group represented by 434 individuals, the *zernovi* species group represented by 385 individuals, and the *I. staneki* group represented by 10 individuals. The data are categorized into six groups on the basis of species and sex. The *rectipennis* species group included 218 females and 216 males. The other species group is *zernovi*, consisting of 188 females and 197 males. A total of 10 *I. staneki* Maran, 1958 individuals (5 males and five females) from the Ilgaz Mountains were included in the analysis as representatives of a distinct group (*I. straubei* species group), which is markedly different from the *zernovi* and *rectipennis* species groups^36,37^. In the *rectipennis* species group, there are 187 nonvirgin individuals and 247 virgin individuals. In *I. staneki*, which is represented by a limited number of individuals, the group includes 10 virgin individuals. The analysis of the *zernovi* species group included 192 nonvirgin individuals and 193 virgin individuals.

### Statistical analyses

To investigate inter- and intra-specific variation in cuticular hydrocarbon (CHC) profiles, raw GC–MS data were processed to construct individual-by-compound matrices. Peak areas (excluding the internal standard, pentadecane) were normalized to control for extraction variation. Compounds consistently absent or near-zero across samples were excluded. Only CHCs detected in ≥ 10 individuals overall and ≥ 5 per sex were retained, resulting in 21 CHCs for males and 18 for females. A subset of n₃ common CHCs was used to construct a shared PCA space. Principal Component Analysis (PCA) was performed separately for males and females, as well as globally across all individuals using the shared CHCs. Data were log-transformed, mean-centered, and scaled to unit variance. Individual metadata (species, species group, sex, mating status) were linked via sample ID. To visualize clustering patterns and between-group variation, 95% confidence ellipses were overlaid using *ggplot2*^56^.

Separate one-way ANOVAs were conducted for PC1–PC5 using Species, Sex, and Mating Status (MS) as predictors, including all two- and three-way interactions. When significant three-way interactions (Species × Sex × MS) were detected, pairwise comparisons among species were performed within each Sex × MS group using the *emmeans* package^57^, with Holm correction applied to control for family-wise error. To assess within-group variability, the betadisper() function from the *vegan* package^58^ was applied to Euclidean distances of PC1–PC5 scores, and differences among species, sexes, and mating status were tested using ANOVA followed by Tukey’s HSD, providing an objective measure of intraspecific CHC heterogeneity. For chemical class-level analyses, CHCs were categorized into six structural classes—n-alkanes, monomethyl- and dimethyl-branched alkanes, alkenes, alkadienes, and unknowns—and their relative abundances (%) were calculated per individual. To examine the effects of sex and mating status on CHC class composition, linear mixed-effects models (LMMs) were fitted using the lmer() function from the *lme4* package^59^, with sex and MS as fixed effects and species as a random effect; pairwise comparisons were conducted using emmeans with Holm correction. All statistical analyses were performed in RStudio using the packages ggplot2, dplyr, tidyr. A map illustrating the locations where the studied *Isophya* species were encountered was generated using QGIS, and samples were collected from the most densely populated areas.

All statistical analyses were performed in RStudio^60^ with *ggplot2*^56^ used for data visualization and *dplyr*^61^ and *tidyr*^62^ for data manipulation and tidying. A map showing the locations where the studied *Isophya* species were encountered was generated using QGIS software^63^ (version 3.36.3; https://qgis.org) based on Shuttle Radar Topography Mission (SRTM) elevation data. The samples were collected from the most densely populated areas.

## Results

### CHC diversity and classification

Analysis of cuticular hydrocarbon (CHC) profiles across *Isophya* species revealed considerable variation in both the number and composition of CHC classes (Supplementary Table S1). In total, 78 CHC compounds were classified into six structural categories: n-alkanes, methyl-branched alkanes (monomethyl), methyl-branched alkanes (dimethyl), alkenes, alkadienes, and unknowns. The number of CHC classes detected per individual varied across species, with most species exhibiting contributions from five or all six classes. Exceptions included *I. staneki*, which showed CHCs belonging to only four major classes (n-alkane, monomethyl-branched alkane, alkadiene, and unknown), and *I. obenbergeri*, where dimethyl-branched alkanes were absent. Quantitatively, n-alkanes were the most dominant CHC class across all species, sexes, and reproductive states, contributing an average of 5–8% to the total CHC profile per individual. Methyl-branched alkanes (monomethyl) also consistently contributed to the profiles of all species, though their relative abundance varied more widely (ranging from ~ 2% to over 16% in *I. nervosa* females). Alkenes and dimethyl-branched alkanes exhibited species- and sex-specific variability. For example, *I. bicarinata* females showed elevated alkene contributions (~ 15%), while dimethyl-branched alkanes were most abundant in *I. zernovi* and *I. autumnalis*. Alkadienes were the least frequently observed and were generally present in low abundance (typically < 3%) or absent in many female samples. Sex-specific comparisons revealed that males tended to exhibit slightly higher levels of monomethyl-branched alkanes and alkenes in several species (e.g., *I. nervosa*, *I. zernovi*), while virgin and nonvirgin individuals differed only marginally in overall CHC class distribution (See Supp.Table 1).

Linear mixed-effects models based on Supp. Table 1 confirmed the class-specific effects of sex and mating status on CHC composition. Alkene proportions were significantly influenced by both sex (F = 8.34, *p* = 0.004) and mating status (F = 6.45, *p* = 0.012), while n-alkanes (F = 6.23, *p* = 0.015) and dimethyl-branched alkanes (F = 5.96, *p* = 0.018) were significantly higher in females. Monomethyl-branched alkanes were also affected by mating status (F = 9.27, *p* = 0.003), with a marginal effect of sex (F = 2.88, *p* = 0.091). No significant sex × mating status interaction was observed in any CHC class, suggesting additive but independent influences.

**Fig. 2:**
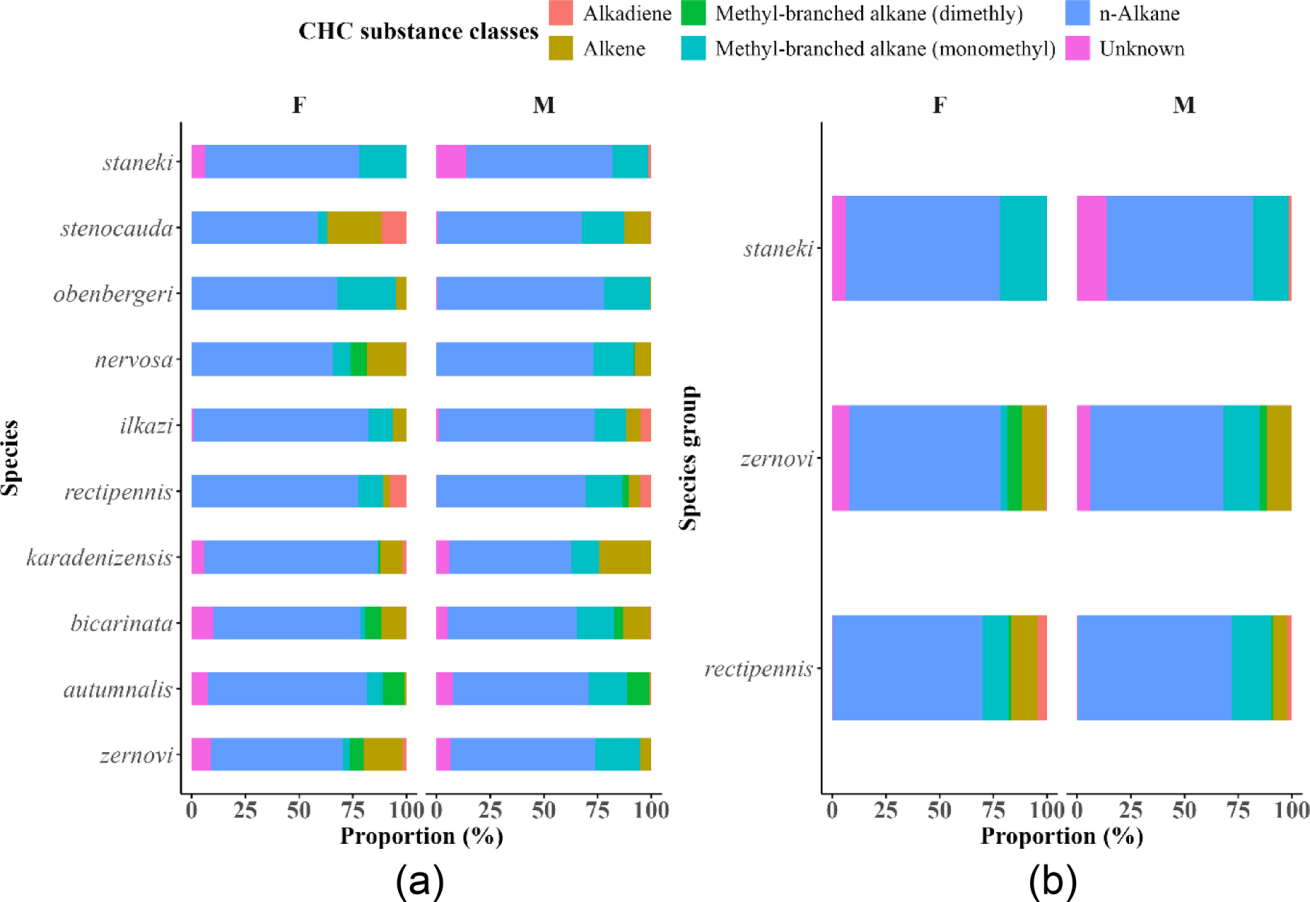
Proportion of cuticular hydrocarbon (CHC) substance classes in different *Isophya* species and three species groups. This figure illustrates the relative proportions of CHC substance classes across male and female individuals within *Isophya* species and species groups. Figure **A** represents the proportional distribution of CHC classes in individual *Isophya* species, while Figure **B** shows the CHC composition in grouped species categories. Each bar segment indicates the proportion of a specific CHC class, highlighting differences in CHC profiles between sexes within each species and group. The CHC classes include alkanes, alkenes, methyl-branched alkanes (dimethyl and monomethyl), unknown compounds, and n-alkanes, providing insights into the chemical diversity and potential ecological or physiological adaptations of *Isophya* species.

**Fig. 3:**
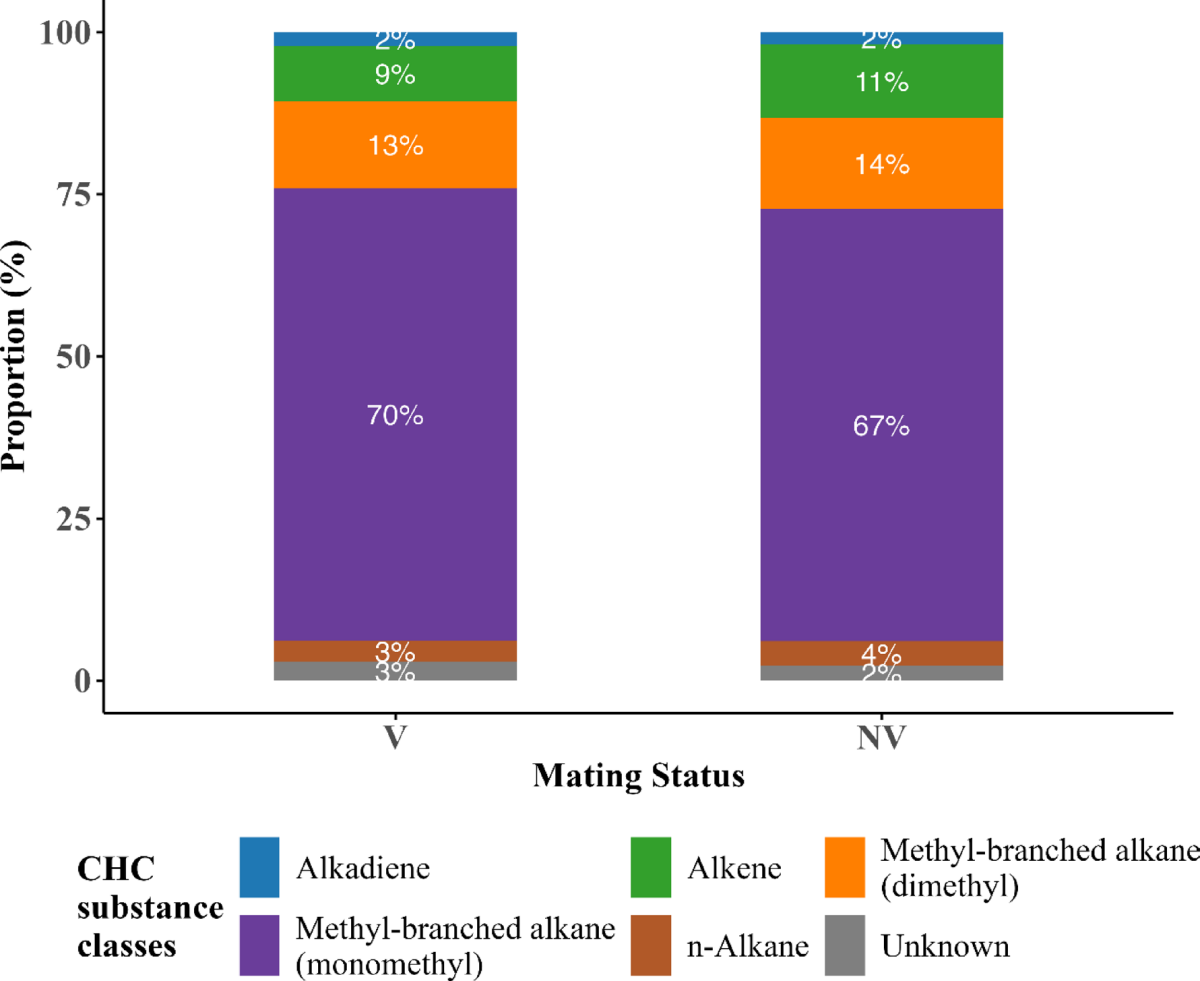
Proportions of cuticular hydrocarbons (CHCs) in unmated (V) and mated (NV) individuals belonging to the *Isophya zernovi* and *Isophya rectipennis* species groups. The bars represent different classes of CHCs, with each class indicated by a distinct color in the legend. The percentages within the bars indicate the relative contribution of each CHC class to the total composition.

**Fig. 4:**
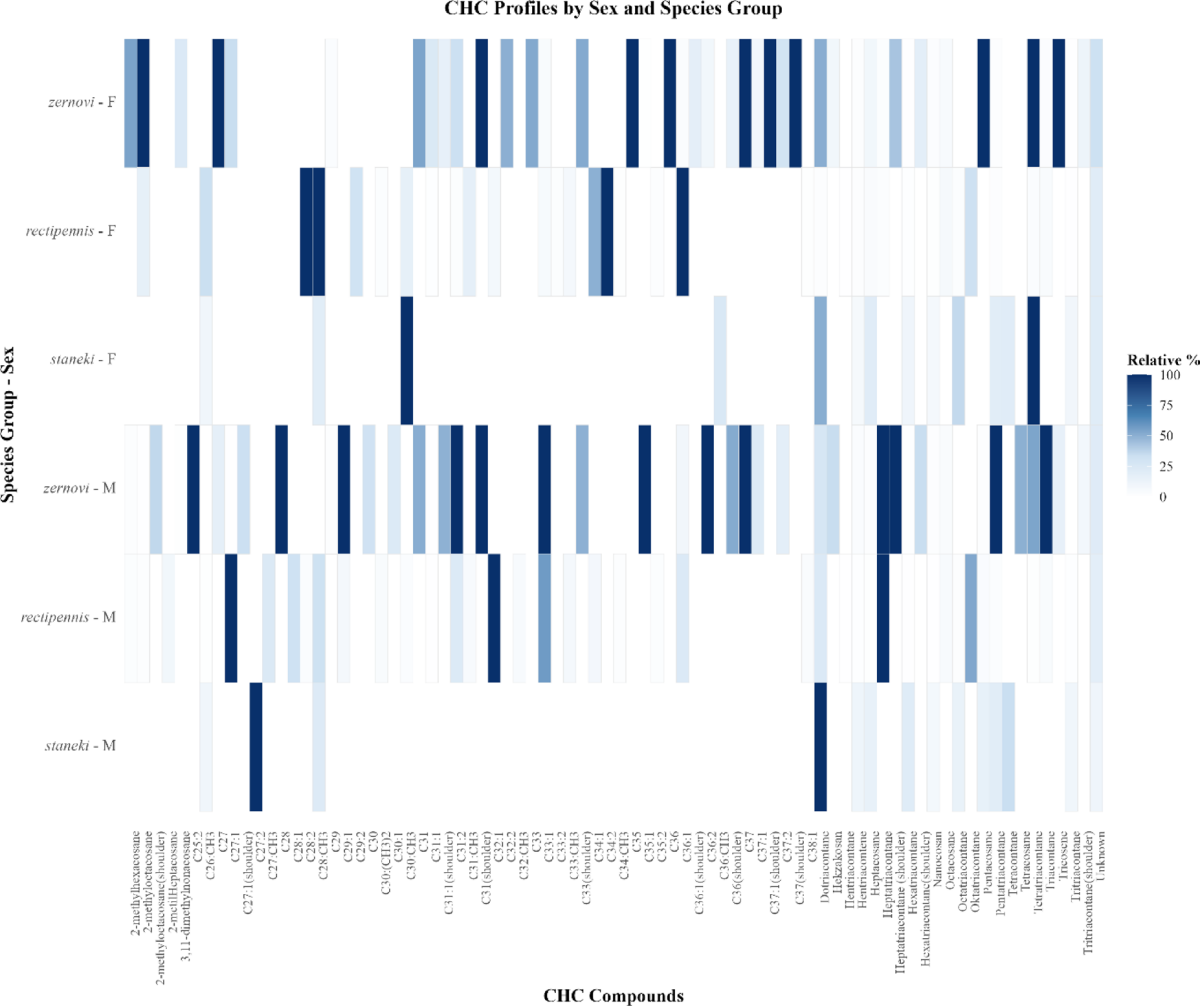
Heatmap of cuticular hydrocarbon (CHC) profiles across different *Isophya* species groups and sexes. The x-axis represents individual CHC compounds, whereas the y–axis represents species groups separated by sex. The color gradient indicates the relative percentage of each compound, with darker shades representing higher relative abundances. Species and sex designations include *zernovi*, *rectipennis*, and *staneki*, denoted by “M” (male) and “F” (female). This visualization highlights the variation in CHC composition across species and sexes, providing insight into potential chemotaxonomic differences.

On average, n-alkanes dominated the CHC profiles across all species, sexes, and mating statuses, contributing between 5 and 8% per individual (Table 1; Figs. 2, 3 and 4). For example, n-alkanes constituted 69.7% of the CHC profile in *I. rectipennis* females and 71.6% in males, while *I. staneki* males and females exhibited 68.0% and 71.7%, respectively. Similarly, *I. zernovi* males showed a notably higher proportion of monomethyl-branched alkanes (17.2%) compared to females (3.29%). These values highlight both species- and sex-specific biases in CHC expression. Methyl-branched alkanes (monomethyl) were consistently present, but with wider interspecific and sex-specific variation—for instance, contributing over 16% in *I. nervosa* females but as low as ~ 2% in *I. stenocauda* males. Alkenes showed pronounced species-level differences, with *I. bicarinata* females exhibiting elevated values (~ 15%), while dimethyl-branched alkanes were especially abundant in *I. autumnalis* and *I. zernovi* (Fig. 2).

**Table 1:** Distribution of cuticular hydrocarbon (CHC) substance classes and their proportions (%) among males (M) and females (F) of three *Isophya* species groups (*I. rectipennis*, *I. zernovi*, and *I. staneki*). The CHC classes identified include alkadienes, alkenes, methyl-branched alkanes (dimethyl and monomethyl), *n*-alkanes, and unknown compounds.

Species group	CHC substances classes	Sex	Proportion (%)
*rectipennis*	Alkadiene	F	4.43
*rectipennis*	Alkadiene	M	2.13
*rectipennis*	Alkene	F	12.1
*rectipennis*	Alkene	M	6.78
*rectipennis*	Methyl-branched alkane (dimethly)	F	1.44
*rectipennis*	Methyl-branched alkane (dimethly)	M	0.612
*rectipennis*	Methyl-branched alkane (monomethy)	F	12.1
*rectipennis*	Methyl-branched alkane (monomethy)	M	18.4
*rectipennis*	Unknown	F	0.186
*rectipennis*	Unknown	M	0.437
*rectipennis*	n-Alkane	F	69.7
*rectipennis*	n-Alkane	M	71.6
*zernovi*	Alkadiene	F	1.05
*zernovi*	Alkadiene	M	0.369
*zernovi*	Alkene	F	10.6
*zernovi*	Alkene	M	11.3
*zernovi*	Methyl-branched alkane (dimethly)	F	6.51
*zernovi*	Methyl-branched alkane (dimethly)	M	3.26
*zernovi*	Methyl-branched alkane (monomethy)	F	3.29
*zernovi*	Methyl-branched alkane (monomethy)	M	17.2
*zernovi*	Unknown	F	8.2
*zernovi*	Unknown	M	6.16
*zernovi*	n-Alkane	F	70.3
*zernovi*	n-Alkane	M	61.7
*staneki*	Alkadiene	M	1.54
*staneki*	Methyl-branched alkane (monomethy)	F	21.9
*staneki*	Methyl-branched alkane (monomethy)	M	16.5
*staneki*	Unknown	F	6.36
*staneki*	Unknown	M	14
*staneki*	n-Alkane	F	71.7
*staneki*	n-Alkane	M	68

The table highlights the variation in CHC profiles between sexes within each species group, which can indicate potential differences in physiological roles, ecological interactions, or mating signals.

CHC substance class composition varied between virgin and non-virgin individuals (Fig. 3). In virgin individuals (V), *n*-alkanes constituted the largest proportion of CHC profiles (70%), followed by alkenes (11%), monomethyl-branched alkanes (8%), and dimethyl-branched alkanes (4%). In contrast, non-virgin individuals (N) showed a slightly lower proportion of *n*-alkanes (67%) and alkenes (9%), but a notably higher proportion of monomethyl-branched alkanes (18%), suggesting a potential enrichment of methyl-branched hydrocarbons after mating. Alkadienes were similarly rare in both groups (3% in virgins, 1% in non-virgins), while the proportion of unknown CHCs remained low and comparable (4% in virgins, 3% in non-virgins). These findings indicate that mating status is associated with subtle shifts in the CHC class composition, particularly in the abundance of methyl-branched compounds.

Representative GC–MS chromatograms illustrating CHC peak profiles of male and female individuals from *I. zernovi*, *I. rectipennis*, and *I. staneki*, each representing one of three species groups, are provided in Supplementary Fig. 2. To further explore sex- and species-specific differences in cuticular hydrocarbon (CHC) composition, we visualized the relative percentage of individual CHC compounds across males and females of *I. zernovi*, *I. rectipennis*, and *I. staneki* using a *heatmap* (Fig. 4). The plot highlights striking inter- and intraspecific variation in CHC profiles, with noticeable differences in both compound richness and relative abundance patterns between sexes and species groups. In general, *I. rectipennis* males exhibited a broader distribution of CHC compounds with relatively higher within-group heterogeneity compared to *I. staneki* and *I. zernovi*. Conversely, *I. staneki* males showed a more restricted set of CHC compounds, consistent with their lower dispersion in multivariate space. Female profiles also reflected species-specific differentiation, with *I. zernovi* females displaying a greater number of abundant CHCs relative to the other two species. Several CHCs, such as C31:1, 2-methyltriacontane, and unknown-1, were detected across most groups but differed in relative intensity, suggesting shared yet differentially regulated components. In contrast, some compounds (e.g., C29:2, C27:1) were either absent or present in very low proportions in certain sex–species combinations, indicating possible group-specific roles in signaling or structural functions.

### Principal component analysis (PCA), MANOVA and univariate ANOVAs

MANOVA revealed significant multivariate effects of *Species*, *Sex*, and *Mating Status* on CHC profiles (all *p* < 0.001). Subsequent univariate ANOVAs showed that *Species* significantly influenced all five principal components (e.g., PC1: F = 20.25, *p* < 0.001), while *Sex* had a particularly strong effect on PC1 (F = 695.52, *p* < 0.001) and PC4 (F = 21.95, *p* < 0.001). *Mating status* significantly affected PC1 (F = 13.87, *p* < 0.001), PC2 (F = 10.22, *p* = 0.001), and PC5 (F = 5.78, *p* = 0.016). Interaction terms were also significant, including the three-way interaction (Species × Sex × Mating Status) across PCs (e.g., PC1: F = 7.44, *p* < 0.001). Univariate ANOVAs were performed on the first five principal components (PC1–PC5) using *Species*, *Sex*, and *Mating Status (MS)* as predictors, including all two-way and three-way interactions. The results are presented in Table 2. The main effects of *Species* and *Sex* were statistically significant for all five PCs, except for the effect of *Sex* on PC5 (*p* = 0.892). The main effect of *Mating Status* was significant for PC3 and PC5. Significant two-way interactions were detected for *Species* × *Sex*, *Species* × *MS*, and *Sex* × *MS* in multiple PCs. The three-way interaction (*Species* × *Sex* × *MS*) was significant for PC1 to PC5.

**Table 2:** Results of univariate ANOVAs for principal components (PC1–PC5).

	PC1		PC2		PC3		PC4		PC5	
Effect	F	*p*	F	*p*	F	*p*	F	*p*	F	*p*
Species	916.370	** < 0.001**	997.580	** < 0.001**	483.090	** < 0.001**	106.630	** < 0.001**	148.370	** < 0.001**
Sex	1422.410	** < 0.001**	187.460	** < 0.001**	202.910	** < 0.001**	1333.630	** < 0.001**	0.020	0.892
MS	0.980	0.322	1.470	0.226	14.350	** < 0.001**	0.760	0.383	59.830	** < 0.001**
Species:Sex	60.770	** < 0.001**	17.260	** < 0.001**	56.180	** < 0.001**	92.830	** < 0.001**	42.600	** < 0.001**
Species:MS	4.490	** < 0.001**	4.090	** < 0.001**	4.440	** < 0.001**	7.840	** < 0.001**	11.680	** < 0.001**
Sex:MS	3.460	0.063	7.240	**0.007**	4.650	**﻿0.031﻿**	0.170	0.684	8.600	**0.003**
Species:Sex:MS	4.840	** < 0.001**	3.060	**0.002**	2.840	**0.004**	5.530	** < 0.001**	11.790	** < 0.001**

Each PC was analyzed using a three-way ANOVA with Species, Sex, and Mating Status (MS) as fixed factors. F-values and associated *p* values are presented for main effects and their interactions. Significant three-way interactions (Species × Sex × MS) indicate that species differences vary depending on both sex and mating status.

Significant values are in bold

**Fig. 5:**
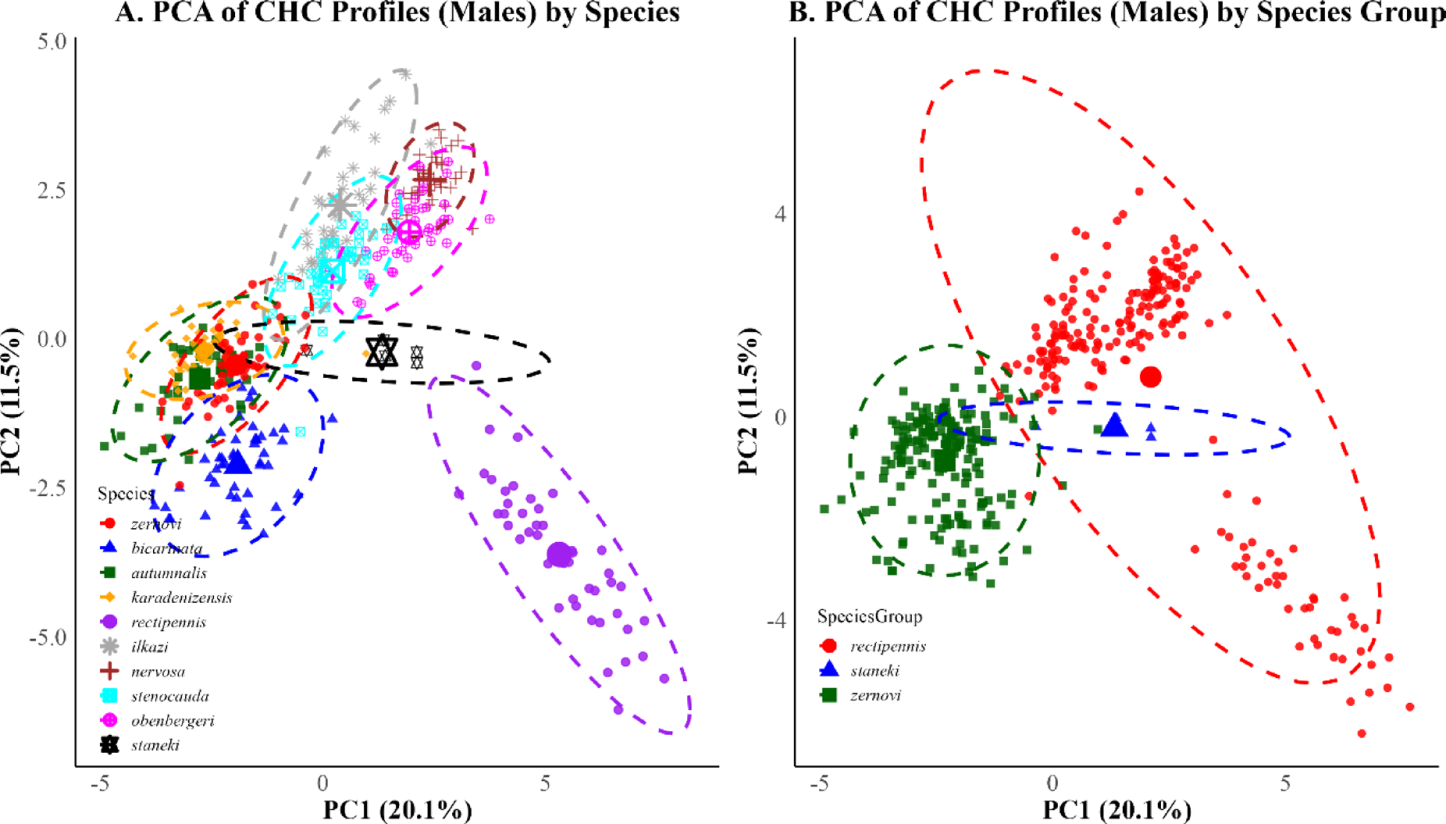
Principal Component Analysis (PCA) of cuticular hydrocarbon (CHC) profiles in male *Isophya* individuals. (**A**) PCA plot showing clustering by species. Members of the *zernovi* group (except *I. bicarinata*) exhibit high overlap and tight clustering, whereas species in the *rectipennis* group display broader dispersion, with *I. rectipennis* forming a clearly separated cluster. *I. staneki* is positioned distinctly from both groups. (**B**) PCA plot showing clustering by species group. The *zernovi* group forms a compact cluster with minimal dispersion, while the *rectipennis* group is more broadly distributed. The *staneki* remains clearly separated. Ellipses represent 95% confidence intervals.

**Fig. 6:**
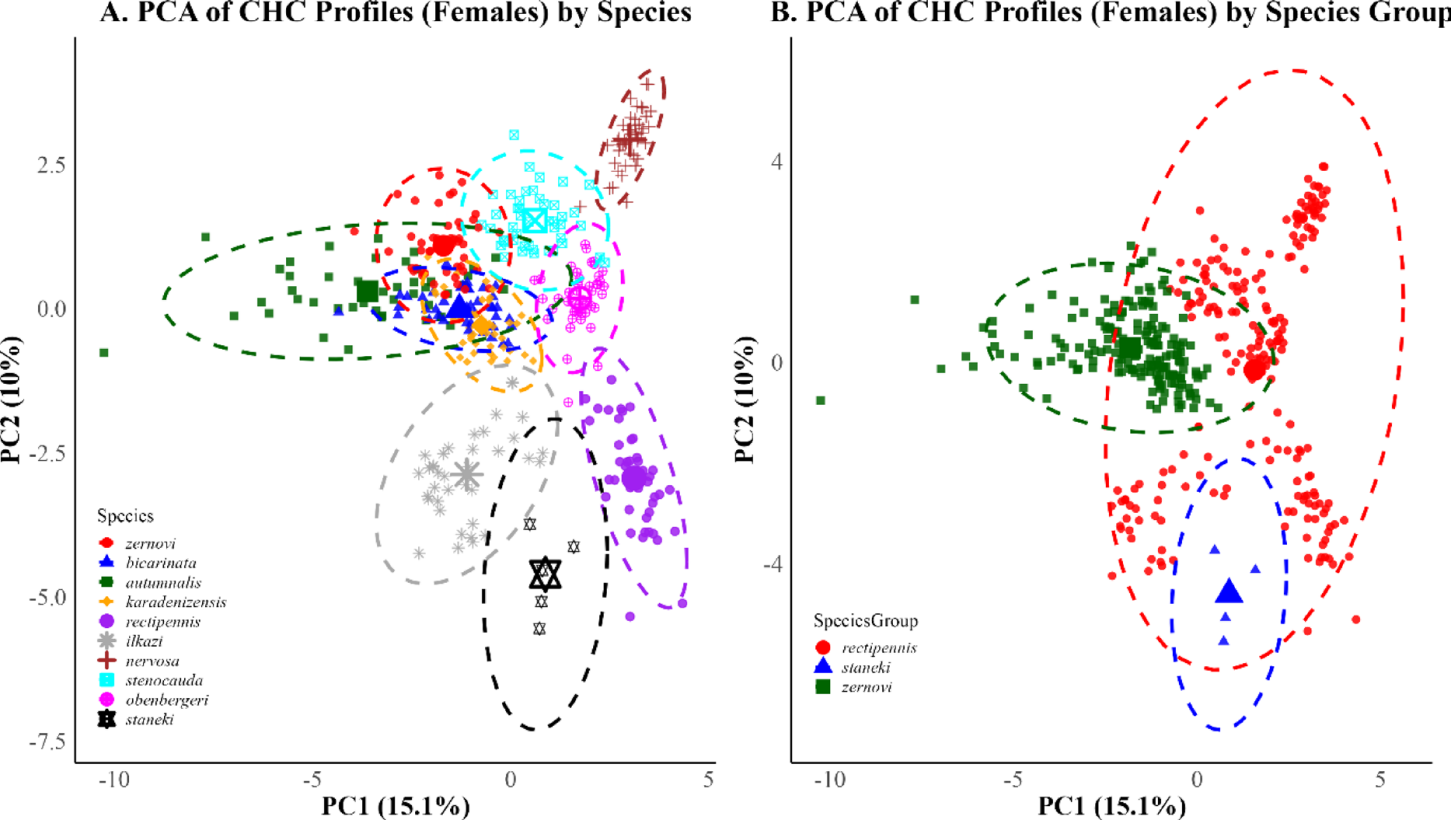
Principal Component Analysis (PCA) of cuticular hydrocarbon (CHC) profiles in female *Isophya* individuals. (**A**) PCA plot showing clustering by species. As in males, *zernovi* group species display tight clustering within a narrow CHC space, whereas *rectipennis* group species are more broadly dispersed. *I. staneki* appears clearly separated from both groups. (**B**) PCA plot showing clustering by species group. The *zernovi* group again forms a compact cluster, while the *rectipennis* group is spread across a wider area in PC space. The *staneki* is distinctly positioned apart from the others. Ellipses represent 95% confidence intervals.

PCA plots for PC1 and PC2 illustrate the overall distribution of CHC profiles by sex and mating status. In males (Fig. 5), clustering patterns were apparent both at the species and species group levels. In females (Fig. 6), similar groupings emerged, although with greater overlap. However, no significant effects of these factors were detected on the first two principal components (two-way ANOVA: all *p* > 0.1). However, ANOVA results on the first five principal components revealed significant effects of mating status on PC3 and PC5 (Table 2), indicating that variation related to reproductive condition is captured in the subtler axes of CHC variation rather than in the primary components.

To identify which principal components (PCs) contributed to the significant multivariate effects, separate ANOVAs were conducted on PC1–PC5 using *Species*, *Sex*, and *Mating Status (MS)* as predictors, including all interaction terms. For PCs with a significant three-way interaction (*Species* × *Sex* × *MS*), pairwise comparisons among species were performed within each combination of *Sex* and *MS* using the *emmeans()* function with *Holm* correction (Supp. Table 2). These comparisons revealed significant differences among species across several sex-by-mating status combinations and principal components.

### CHC compounds contributing to principal components

The top five CHC contributors for each of the first five principal components (PC1–PC5), along with their absolute loading values and compound classes, are listed in Supp. Table 3. For PC1, the strongest contributors included *n*-alkanes such as Heptacosane and Pentacosane, as well as methyl-branched alkanes like 2-methyloctacosane and 2-methylhexacosane. PC2 was mainly driven by C38:1 (alkene), C34:CH3 (methyl-branched alkane), and C35:2 (alkadiene). PC3 featured prominent contributions from methyl-branched alkanes (C26:CH3, C28:CH3) and *n*-alkanes (Hentriacontane, Triacontane). PC4 was characterized by high contributions from *n*-alkanes including Nanocosan, Triacontane, and Tetratriacontane, while PC5 was influenced by both alkenes (C31:1) and branched alkanes (C30:(CH3)2, C34:CH3).

### Intraspecific dispersion and betadisper results

To address potential subjectivity in interpreting visual separation and overlap among species in PCA plots, we employed statistical testing of within-group variability using the betadisper function from the *vegan* package, based on Bray–Curtis distances. This analysis quantifies group-wise dispersion (i.e., the average distance of samples to their group centroid), allowing for objective comparison of intraspecific variation across species.

The results revealed a significant difference in dispersion among species (ANOVA: F = 59.65, *p* < 0.001), confirming that species vary in the extent of individual variability in CHC profiles (Figs. 5A, 6A). *I. staneki* exhibited significantly broader dispersion than several other species, such as *I. autumnalis* (difference = − 0.127, *p* < 0.001), *I. bicarinata* (− 0.186, *p* < 0.001), and *I. karadenizensis* (− 0.243, *p* < 0.001), supporting the earlier visual impression of broader within-species spread (see Supp. Table 3for full pairwise comparisons). Conversely, species such as *I. stenocauda* and *I. ilkazi* displayed narrower dispersions, which were not significantly different from one another (*p* = 1.000). *I. rectipennis* exhibited significantly broader multivariate dispersion compared to most other species—such as *I. autumnalis* (− 0.057, *p* < 0.001), *I. bicarinata* (− 0.116, *p* < 0.001), *I. karadenizensis* (− 0.173, *p* < 0.001), *I. ilkazi* (− 0.065, *p* < 0.001), *I. stenocauda* (− 0.060, *p* < 0.001), and *I. zernovi* (− 0.082, *p* < 0.001)—but its dispersion was not significantly different from that of *I. staneki*, *I. nervosa*, or *I. obenbergeri*.

Although the initial PCA interpretation mentioned apparent overlap and separation, we now clarify that our statistical analysis formally supports variation in dispersion and thus justifies the differential clustering patterns observed. Additionally, while the number of individuals per species was not equal, we note that betadisper is relatively robust to modest sample size differences, and caution was taken in interpreting clusters with low *n*.

We also tested group dispersions for sex and mating status. No significant difference in dispersion was found between males and females (*F* = 2.41, *p* = 0.121), indicating comparable within-sex variability in CHC expression. However, mating status had a significant effect (*F* = 7.61, *p* = 0.006), with nonvirgin individuals exhibiting significantly greater dispersion compared to virgins (Tukey’s HSD: diff = 0.0139, adjusted *p* = 0.0059), suggesting increased variability in CHC profiles post-mating. This pattern is visually supported in the PCA plot (Supp. Figure 3), where nonvirgin individuals of both sexes appear more dispersed compared to virgins, although there is no distinct separation along the first two principal components.

In addition to species-level comparisons, we assessed multivariate dispersion across species groups. In males, species groups differed significantly in within-group dispersion (ANOVA: F = 20.96, *p* < 0.001), with the *rectipennis* group displaying broader dispersion than both the *staneki* (*p* < 0.001) and *zernovi* (*p* < 0.001) groups (Fig. 5B). The *staneki* group was also significantly more dispersed than the *zernovi* group (*p* = 0.001), supporting the visual impression of a tight *zernovi* group cluster. A similar pattern emerged in females (ANOVA: F = 10.78, *p* < 0.001), with significantly higher dispersion in the *rectipennis* group compared to staneki (*p* < 0.001), while no significant difference was detected between *rectipennis* and *zernovi* (*p* = 0.97) (Fig. 6B). However, the *zernovi* group again clustered more tightly than the *staneki* group (*p* < 0.001).

## Discussion

This study revealed significant variability in CHC profiles across *Isophya* species, with certain CHC classes predominating and others remaining at low levels. These results indicate that CHC composition differs according to species identity and sex, potentially reflecting underlying ecological or physiological differences. While such patterns may align with adaptive explanations, our study does not directly test functional hypotheses. Therefore, any interpretations regarding ecological or reproductive roles should be considered preliminary and warrant further investigation.

### CHC diversity and species-specific patterns

The remarkable diversity of CHCs serves critical biological functions, such as communication and desiccation resistance, underscoring their importance in the evolutionary success of insects^21,64–66^. In the genus *Isophya*, 78 unique CHC compounds were identified and classified into six distinct types, including n-alkanes, species^67^. This dominance of n-alkanes is consistent with their known role in waterproofing and environmental adaptation, as observed in other Orthopteran taxa, such as *Pamphagus elephas*, where n-alkanes constitute more than 72% of CHCs in both sexes^68^. The variation in CHC profiles reflects both environmental adaptation and evolutionary pressures that shape these chemical signatures^69^. Notably, *I. bicarinata* and *I. staneki* exhibited distinct CHC diversity, with 36 and 17 CHC types, respectively. These species-specific chemical profiles may correspond to differences in ecological niches or reproductive contexts, although functional roles were not directly tested.

Such CHC richness is comparable to that reported in the butterfly *Bicyclus anynana*, where 103 distinct compounds were identified across body parts, sexes, and ages^70^. However, unlike *B. anynana*, which was analyzed by dissecting body parts, our study was based on whole-body CHC extracts, and thus does not capture potential intra-individual spatial variation. In contrast, the zaprochiline tettigoniid *Kawanaphila nartee* exhibited only 23 compounds, without clear class differentiation^49^. This variation manifested in compound types; for instance, dimethyl-branched alkanes were enriched in some species, such as *I. zernovi*, but absent in others, like *I. obenbergeri.* In *K. nartee*, sexual dimorphism was limited to chain length differences, with females having longer-chain CHCs. For example, CHC class distributions differed notably between species and sexes: *I. staneki* females exhibited notably high proportions of monomethyl-branched alkanes, while *I. rectipennis* and *I. zernovi* females were characterized by higher n-alkane percentages and moderate alkene content. In males, CHC class proportions were more evenly distributed across species, though *I. staneki* males had higher levels of uncharacterized CHCs grouped under the “Unknown” category. This trend emphasizes the central role of n-alkanes in *Isophya* CHC profiles, suggesting their functional relevance across taxa and contexts.

These patterns are not unique to *Isophya*. Similar CHC class-specific variation has been observed across diverse insect taxa. In Lepidoptera, for instance, Heuskin et al.^70^ demonstrated that specific cuticular compounds varied according to sex, body part, and age, thereby contributing to chemical discrimination. Likewise, in *Macrolophus* plant bug species, Gemeno et al.^71^ found that CHC profiles provided reliable cues for species and sex identification, even under dietary variation. Furthermore, sexual dimorphism in CHC composition has been described in Coleoptera (e.g., *Monochamus scutellatus*), where hydrocarbon profiles vary with sex and feeding status^72^. However, since diet or feeding status was not assessed in our study, we cannot determine whether similar physiological influences contribute to CHC variation in *Isophya*.

The complexity and specificity of *Isophya* CHC profiles are comparable to the findings in the grasshopper genera, *Pamphagus* and *Chorthippus*, where methyl-branch positions and chain lengths significantly influence species and sex differentiation^18,68,73^. For example, *Chorthippus biguttulus* and *C. mollis* separate into species and sexes based on methyl-branched hydrocarbons, with PCA revealing differentiation across principal components^18^.

In *Isophya*, notable differences in CHC composition are observed among species groups (*zernovi*, *rectipennis*, and *staneki*). Certain hydrocarbons, such as C27:1 and C33, show notable variations in abundance. Nakano et al.^4^ underscored the importance of these findings. These authors reported that the migratory locusts *Schistocerca gregaria* and *Locusta migratoria* exhibit highly distinct CHC profiles, with certain hydrocarbons being particularly dominant in each species. Moreover, the CHC profiles of *Schistocerca* and *Locusta*, as described in their study, were chemically more complex, encompassing a diverse range of hydrocarbons, including methyl-branched alkanes and alkenes. This relative chemical simplicity may reflect ecological differences, as *Isophya* species exhibit fewer dominant CHCs compared to other genera, such as acridid grasshoppers *Chorthippus* and *Pamphagus*. Further functional studies are necessary to clarify the ecological significance of these patterns.

Our findings are consistent with and enriched by insights from studies on other Orthopteran taxa, such as *Pamphagus elephas*^68^, *Gryllus firmus*, and *G. pennsylvanicus*^21^. In *P. elephas*, sex-specific CHC variation was observed, with females producing higher levels of trimethylalkanes, suggesting potential reproductive roles for CHCs. Similarly, our results revealed both sexual dimorphism and interspecific divergence in CHC profiles across *Isophya* species. In males, members of the *zernovi* group (excluding *I. bicarinata*) showed high overlap and tight clustering, whereas *rectipennis* group species displayed broader dispersion, with *I. rectipennis* being clearly separated. *I. stenocauda*, *I. ilkazi*, and *I. obenbergeri* formed partially overlapping but distinguishable clusters, and despite *I. obenbergeri* being treated as a subspecies of *I. stenocauda*, its CHC profile showed signs of divergence. In females, the *zernovi* group again exhibited tight clustering, while *rectipennis* group members were more dispersed. Notably, *I. staneki* was clearly separated from all others in both sexes. These patterns suggest that CHC profiles capture both sex-based and species-level differences, with potential links to reproductive isolation and chemical signaling. However, further functional studies are needed to clarify whether CHCs directly mediate mate recognition or speciation processes in *Isophya*. Supporting the taxonomic utility of CHCs, studies on *Gryllotalpa* mole crickets demonstrated that quantitative CHC differences enabled separation of *G. tali* and *G. marismortui* despite qualitative similarity, and that CHCs, when combined with acoustic and chromosomal traits, contributed to robust species delimitation^74,75^. Similarly, our findings reinforce the integrative value of CHC profiles for resolving species boundaries in closely related taxa and cryptic species complexes.

### CHC class distribution and dominance of n-alkanes

Across all the species, n-alkanes were the most abundant CHC class in both sexes, ranging from 56.90% in *I. karadenizensis* males to 81.24% in *I. ilkazi* females. This pattern aligns with previous observations suggesting that n-alkanes can form a strong hydrophobic barrier on the cuticle, which helps regulate moisture and prevent desiccation under variable humidity conditions^32,67,76^. Most of the studied *Isophya* species inhabit the Black Sea region of Turkey, where rainfall and humidity are relatively high, and the green vegetation cover is denser than in other areas. Furthermore, in comparison with species in Acrididae and Pamphagidae, *Isophya* species possess visibly softer and thinner cuticles. This anatomical characteristic may be associated with their habitat preferences, as suggested by Khemaissia et al.^77^ who noted that cuticle thickness could correlate with moisture tolerance. While this observation may help contextualize the high proportion of n-alkanes in *Isophya* CHC profiles, direct testing of such physiological-environmental associations was beyond the scope of the present study.

Research on *Drosophila* species has further elucidated the adaptive significance of CHCs. For example, studies have shown that longer-chain hydrocarbons are associated with increased desiccation resistance, a trait that has evolved in response to environmental pressures^28,78,79^. In *D. suzukii*, seasonal changes in temperature and photoperiod have been linked to alterations in CHC profiles, affecting mating success and indicating a dynamic response to environmental conditions^80^. This adaptability is truly remarkable, as it allows insects to optimize their CHC profiles according to their specific habitats, thus increasing their survival and reproductive success^28,79^. These findings suggest that CHCs may exhibit some plasticity in response to environmental variation. For example, in the *rectipennis* group, n-alkane levels exceeded 70% in both sexes. In contrast, species in the *zernovi* group exhibited more variation, with some species, such as *I. bicarinata* and *I. autumnalis,* having notably higher alkene levels (up to 16%). These interspecific and intersexual differences suggest that ecological constraints may shape the relative composition of CHC classes in a lineage-specific manner.

While our study does not directly test functional responses, the observed differences in CHC profiles between sexes and species in *Isophya* may be influenced by ecological or physiological factors. In line with *Drosophila* studies, these patterns raise the possibility that CHC composition in *Isophya* varies with habitat characteristics; however, this hypothesis requires further experimental investigation to confirm any adaptive significance.

The CHC profiles also exhibit variability in other classes. Alkenes and, to a lesser extent, dimethyl-branched alkanes are more prominent in species inhabiting wetter climates, such as *I. stenocauda* and *I. karadenizensis*. Although the functional roles of these compounds were not examined in this study, previous work has suggested that alkenes may contribute to desiccation resistance. While this function is typically associated with arid environments, it has been proposed that maintaining cuticular integrity and preventing excess water exchange may also be advantageous in humid habitats, where fluctuating microclimatic conditions can influence water balance. The “Unknown” category also shows noticeable variability across species, with *I. stenocauda* and *I. staneki* exhibiting higher proportions than the other studied species. While the role of these unknown compounds remains unclear, they may contribute to microbial resistance or complement water-balance regulation under variable moisture regimes^67^. These variations in other CHC classes, as well as sex-specific and species-specific patterns, highlight the influence of environmental, physiological, and selective pressures.

In our PCA analyses, the primary contributors to PC1–PC5 included long-chain *n*-alkanes (e.g., Heptacosane, Pentacosane), methyl-branched alkanes (e.g., 2-methyloctacosane, C28:CH3, C34:CH3), and alkenes (e.g., C31:1, C38:1), indicating that interspecific and sex-based differences in CHC profiles are shaped by variation in hydrocarbon chain length and branching patterns. While we did not directly investigate their functional roles, similar patterns have been observed in *Drosophila*, where variation in chain-elongated and branched CHCs contributes to sex-specific signaling and mating outcomes, for example, Luo et al.^81^ demonstrated that male-biased long-chain alkenes such as 9-pentacosene and 9-heptacosene reduce female attractiveness when artificially applied, highlighting the behavioral impact of chain-elongated hydrocarbons. In parallel, Dembeck et al.^82^ identified extensive natural genetic variation in *D. melanogaster* CHC profiles, driven by genes involved in fatty acid metabolism and elongation, including previously uncharacterized loci that modulate chain length and composition. Together, these patterns were particularly pronounced in males of *I. staneki*, which exhibited elevated levels of long-chain CHCs and unclassified compounds, suggesting that similar evolutionary forces (e.g., sexual selection, ecological adaptation) may underlie hydrocarbon profile divergence in *Isophya*. Although we did not assess behavioral effects directly, our findings support the hypothesis that both genetic and environmental factors shape involved in sexual communication.

### Sex-based and mating status-dependent differences in CHC profiles

The multivariate analysis of *Isophya* CHC profiles revealed significant effects of species, sex, mating status, and their interactions. Species identity had the strongest overall effect across PCs. This finding is consistent with the literature that emphasizes the role of CHCs in species recognition and sexual selection among insects^55,83^. A significant species-by-sex interaction suggests that CHC profiles vary between males and females in a species-specific manner. While such patterns may be consistent with sex-specific chemical signaling, the current study does not directly assess behavioral outcomes. Notably, sex had a strong effect on PC1, indicating pronounced sexual dimorphism in CHC profiles within *Isophya* species. This finding is consistent with previous studies on *Teleogryllus oceanicus*, where males and females exhibited distinct CHC compositions, likely shaped by sexual selection^84^.

Mating status (MS) contributed to CHC variation, although its overall influence was more restricted compared to the dominant effects of species and sex. Rather than producing a uniform shift across all chemical axes, the influence of mating status appeared selectively expressed in specific components of multivariate variation. Moreover, significant interactions between species, sex, and mating status indicate that a combination of sexual dimorphism, reproductive condition, and species-specific factors may influence the expression of CHCs. This observation parallels findings in sagebrush grig *Cyphoderris strepitans* and the stingless bee *Melipona scutellaris*, where CHC profiles vary with mating history^55,85^.

Moreover, our findings align with those of a study on *D. melanogaster*, where mating status altered CHC profiles through three potential mechanisms, including the transfer of cuticular and seminal hydrocarbons from males during copulation^26,86^. These transferred compounds reduce the female’s attractiveness and modulate her receptivity to future matings. Notably, however, the experimental design in our study differs in an important aspect: in *Isophya*, males transfer a spermatophore consisting of an ampulla and a protein-rich spermatophylax, which females typically consume after copulation^50,87^. In our experimental setup, the consumption of the spermatophylax was prevented to control for nutritional or post-copulatory chemical influences. This distinction may have limited the degree or nature of CHC modulation observed in our study compared to systems like *Drosophila*, where ejaculate-associated compounds are fully transferred and retained.

Likewise, studies on other Diptera, such as *Dasineura oleae,* have described comparable shifts in CHC composition associated with mating status^9^. The significant *species:sex:mating status* interaction suggests that CHC expression is modulated not only by sexual dimorphism and mating history but also by species-specific factors. This pattern is consistent with the dual role of CHCs proposed in previous work^10,64^. While our findings are consistent with such patterns, we emphasize that our data are descriptive, and no functional or mechanistic interpretations can be inferred without additional experimental validation.

Additionally, studies on the meadow grasshopper species *Chorthippus biguttulus* and *C. mollis* have indicated that CHC profiles differ significantly between sexes, with PC2 explaining a substantial portion of the variation between males and females^18^. The observation that PC2 also significantly separated the sexes in our *Isophya* dataset supports the generality of CHC-based sexual dimorphism across Orthoptera. Furthermore, the significant *species:sex* interaction in our results mirrors findings in *Chorthippus*, where CHC divergence is species- and sex-dependent. The parallel between our findings and those in *Chorthippus*, particularly the significant species-by-sex interaction, suggests that sex-related variation in CHC composition may be a broader phenomenon among Orthoptera. However, further research is needed to confirm this in *Isophya*. In summary, our PCA results demonstrate that CHC composition in *Isophya* is significantly influenced by species identity, sex, and mating status.

Alkenes and alkadienes, though less dominant, show interesting patterns related to sex differences. In species such as *I. stenocauda*, females have exceptionally high proportions of alkenes and alkadienes, whereas males present significantly lower percentages. These unsaturated hydrocarbons, due to their higher permeability to water vapor, have been proposed to play roles beyond moisture retention, possibly in chemical signaling or mate recognition^10^. Although this study does not directly test these roles, the observed sex-based differences in alkene and alkadiene abundance may be consistent with such functions, as reported in other insect taxa^67^. Methyl-branched alkanes, particularly monomethyl-branched alkanes, are prominent in several species, with males often having higher levels than females. For example, *I. zernovi* males presented 20.89% monomethyl-branched alkanes, whereas females presented 2.87% monomethyl-branched alkanes. This dimorphism suggests that methyl-branched hydrocarbons could function in intersexual communication, potentially as pheromonal signals^88^. The presence of these compounds in relatively humid environments may therefore support dual functions—enhancing mating behavior through distinct scent profiles, while also providing a protective barrier that stabilizes cuticular permeability^10^.

Sexual dimorphism and post-mating effects were also more structurally diverse in *Isophya*. For example, dimethyl-branched alkanes were notably enriched in *I. staneki* females, while nonvirgin individuals across several groups showed increased monomethyl-branched alkanes. These findings underscore that CHC expression in *Isophya* is not only modulated by mating history but also strongly influenced by phylogenetic lineage and habitat-specific factors—each species group occupying ecologically distinct zones that may drive CHC divergence through selection. However, their specific functions remain to be tested in *Isophya*. Similar sex-specific differences in CHC abundance have been reported in White-spotted Sawyer *Monochamus scutellatus*, where both sexes possess the same hydrocarbon compounds. Yet, their relative proportions differ significantly—particularly in monoenes and methyl-branched alkanes^72^. Moreover, the quantity of CHCs increases markedly in maturation-fed females compared to unfed ones, suggesting that physiological states such as nutritional status can modulate CHC expression. These results reinforce the idea that CHC profiles are shaped not only by sex and species but also by ecological and physiological conditions. CHC composition, shaped by species identity, sex, and mating status, appears to serve dual functions in sexual selection and ecological adaptation, as also proposed in prior studies across Orthoptera and Diptera.

In addition to differences in CHC class composition across species and sexes, we also found notable variation in within-group CHC dispersion. Our betadisper analysis revealed that particular species, such as *I. staneki* and *I. rectipennis*, exhibited significantly broader intraspecific dispersion compared to others like *I. autumnalis*, *I. stenocauda*, or *I. ilkazi*. This elevated variability may reflect greater ecological plasticity or developmental heterogeneity in CHC expression. Notably, the rectipennis species group also displayed higher within-group dispersion compared to other species groups in both sexes, reinforcing the idea that lineage-specific traits and habitat heterogeneity may shape CHC variability. These findings are consistent with^89^, who highlighted that environmental factors, such as diet, microclimate, and humidity, as well as life stage and physiological state, can significantly modulate CHC expression and increase within-species variation in insects. Conversely, the tightly clustered CHC profiles in species like *I. zernovi* may indicate more canalized or environmentally constrained hydrocarbon expression. Furthermore, mating status significantly influenced CHC dispersion: nonvirgin individuals showed greater within-group variability than virgins, suggesting that post-mating physiological or chemical changes contribute to increased individual heterogeneity. These results highlight that, beyond categorical differences, CHC profiles also vary in the extent of individual-level variability—a feature that may play a role in fine-tuning mate choice or signaling robustness in natural populations. Overall, while these results suggest intriguing patterns, further experimental studies are needed to clarify whether these CHC classes contribute to mating behavior, chemical communication, or environmental adaptation.

### Evolutionary and ecological﻿ ﻿implications

The dominance of n-alkanes across *Isophya* species suggests a conserved role in desiccation resistance. At the same time, the diversity of methyl-branched alkanes and alkenes may reflect evolutionary divergence in chemical signaling. These findings highlight the ecological relevance of CHCs in species differentiation and adaptation among Orthoptera. As in other insects, *Isophya* CHCs form a hydrophobic cuticular barrier critical for water retention, especially in variable environments. The relative abundance of long-chain and branched hydrocarbons in *Isophya* aligns with patterns seen in montane and arid-adapted insects, where such compounds enhance moisture retention. These results support previous findings emphasizing the dual role of CHCs in both adaptation and communication.

*Isophya* species typically inhabit humid regions, such as the Black Sea and the Balkan highlands. However, some populations, such as *I. sikorai* and *I. savignyi* from the arid regions, emerge during rainy seasons, suggesting plasticity in CHC expression in response to seasonal moisture—a phenomenon also observed in other insects like rice planthoppers. Distinct CHC compositions among species likely reflect habitat associations. For example, *I. karadenizensis* shows high n-alkane content, which may contribute to regulating cuticular permeability in humid areas rather than strictly conserving water as in xeric conditions. Conversely, *I. stenocauda* females exhibit elevated alkenes, possibly indicating a balance between desiccation and signaling, while *I. rectipennis* females have notable levels of alkadienes that may serve habitat- or mating-related functions.

Species group-level CHC patterns support the role of ecological differentiation: *staneki* group in montane steppe, *zernovi* in humid forest edges, and *rectipennis* in transitional zones. These associations may drive selection on cuticular chemistry. Additionally, as observed by^9^ in the gall midge *Dasineura oleae*, some CHC components may originate from ecological exposure or dietary uptake during development.

The greatest CHC diversity in *I. bicarinata* and the lowest in *I. staneki* reflect broader trends, and suggesting that ecological factors influence chemical variation. The consistent dominance of n-alkanes across species—over 60% in most cases—mirrors findings in other grasshoppers (e.g., *Chorthippus*) and may represent an adaptive trait for desiccation resistance. In sum, our research revealed that *Isophya* CHC profiles are shaped by a combination of species identity, habitat conditions, and potentially seasonal environmental factors. While patterns suggest ecological plasticity, further research is needed to determine the adaptive significance of these patterns.

## Conclusion

This study highlights the ecological and evolutionary relevance of cuticular hydrocarbons (CHCs) in *Isophya* species, revealing significant interspecific variation, sexual dimorphism, and mating status-dependent differences in CHC profiles. The dominance of n-alkanes and the species-specific distribution of methyl-branched alkanes and alkenes suggest adaptive roles in desiccation resistance and chemical communication. While most *Isophya* species inhabit humid environments, some populations in arid regions exhibit CHC traits potentially shaped by seasonal moisture availability, indicating ecological plasticity. Distinct CHC compositions across species and species groups appear linked to habitat differences, reflecting selection pressures on cuticular chemistry. However, these functional interpretations remain speculative without validation through behavioral or physiological studies. Notably, the univoltine life cycle of *Isophya* reduces the likelihood of strong seasonal effects on CHC expression, though dietary influences across habitats remain an open question.

In conclusion, the variation in CHCs in *Isophya* offers valuable insights into the chemical diversification of Orthoptera. However, these functional interpretations remain speculative and require validation through behavioral or physiological studies. Notably, the univoltine life cycle of bushcrickets, such as the genera *Isophya* and *Poecilimon,* reduces the likelihood of strong seasonal effects on CHC expression. However, dietary influences across habitats remain an open question.

## Supplementary Information

Below is the link to the electronic supplementary material.

Supplementary Material 1

Supplementary Material 2

Supplementary Material 3

Supplementary Material 4

Supplementary Material 5

Supplementary Material 6

Supplementary Material 7

## Data Availability

The dataset analyzed during the current study is available from the corresponding author upon reasonable request.
