# Supplementary figures and images for: Cuticular hydrocarbon profiles in plump bush crickets vary according to species, sex and mating status
Source: Sci Rep. 2025 Sep 26;15:33233. doi: 10.1038/s41598-025-17544-7 (PMC12475074; doi:10.1038/s41598-025-17544-7)

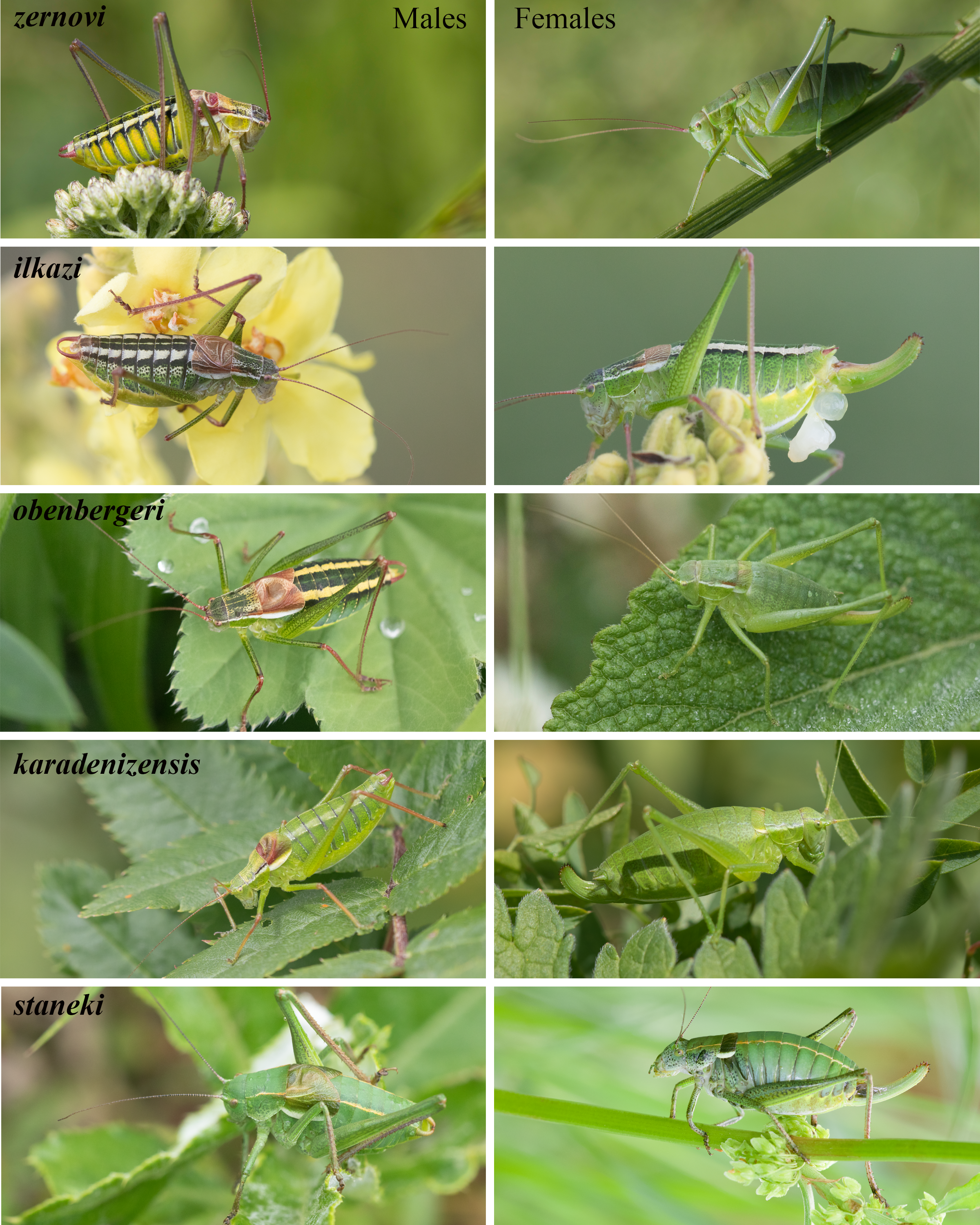

Supplement: Supplementary file 1 — Supplementary Material 1 [file 41598_2025_17544_MOESM1_ESM.tif]

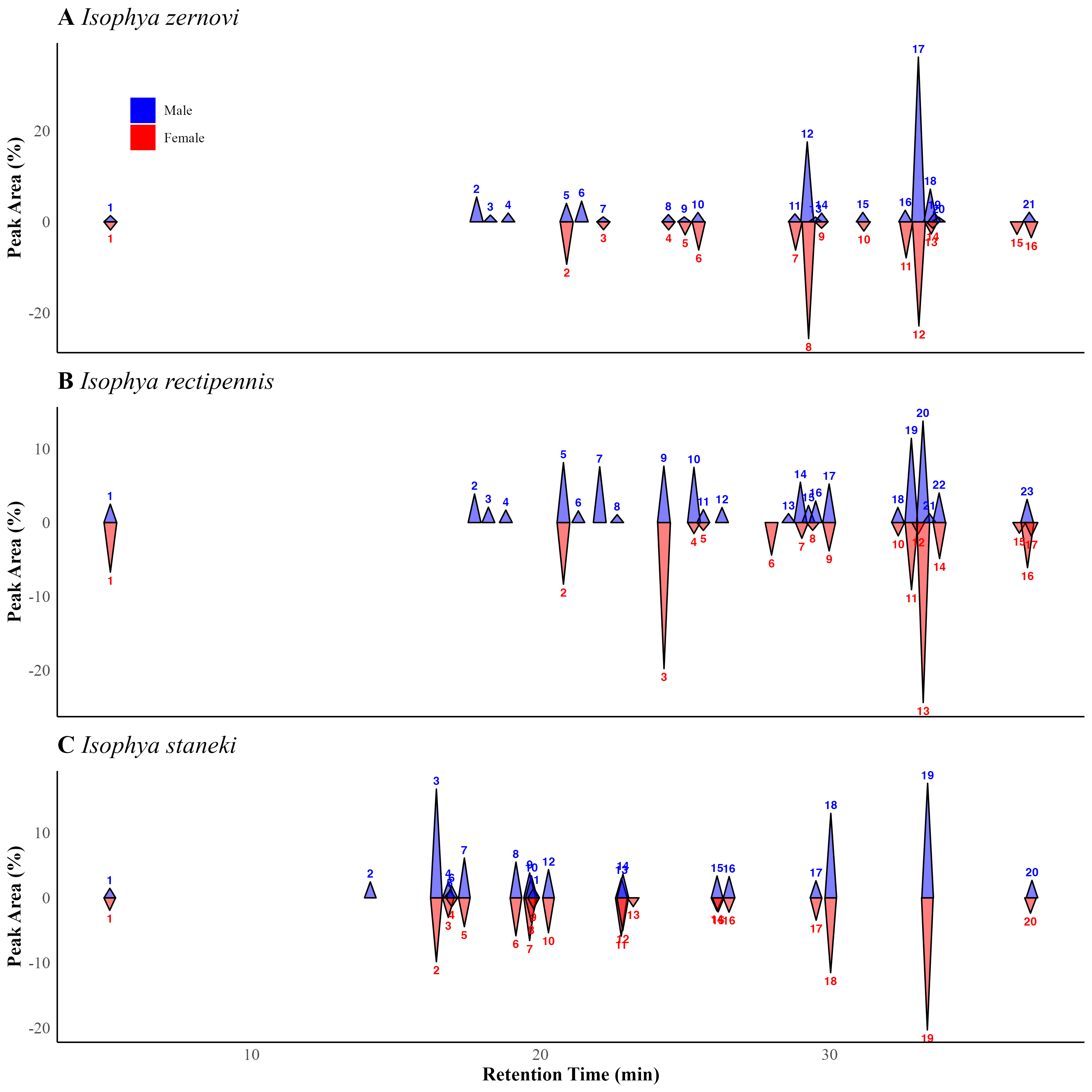

Supplement: Supplementary file 2 — Supplementary Material 2 [file 41598_2025_17544_MOESM2_ESM.tiff]

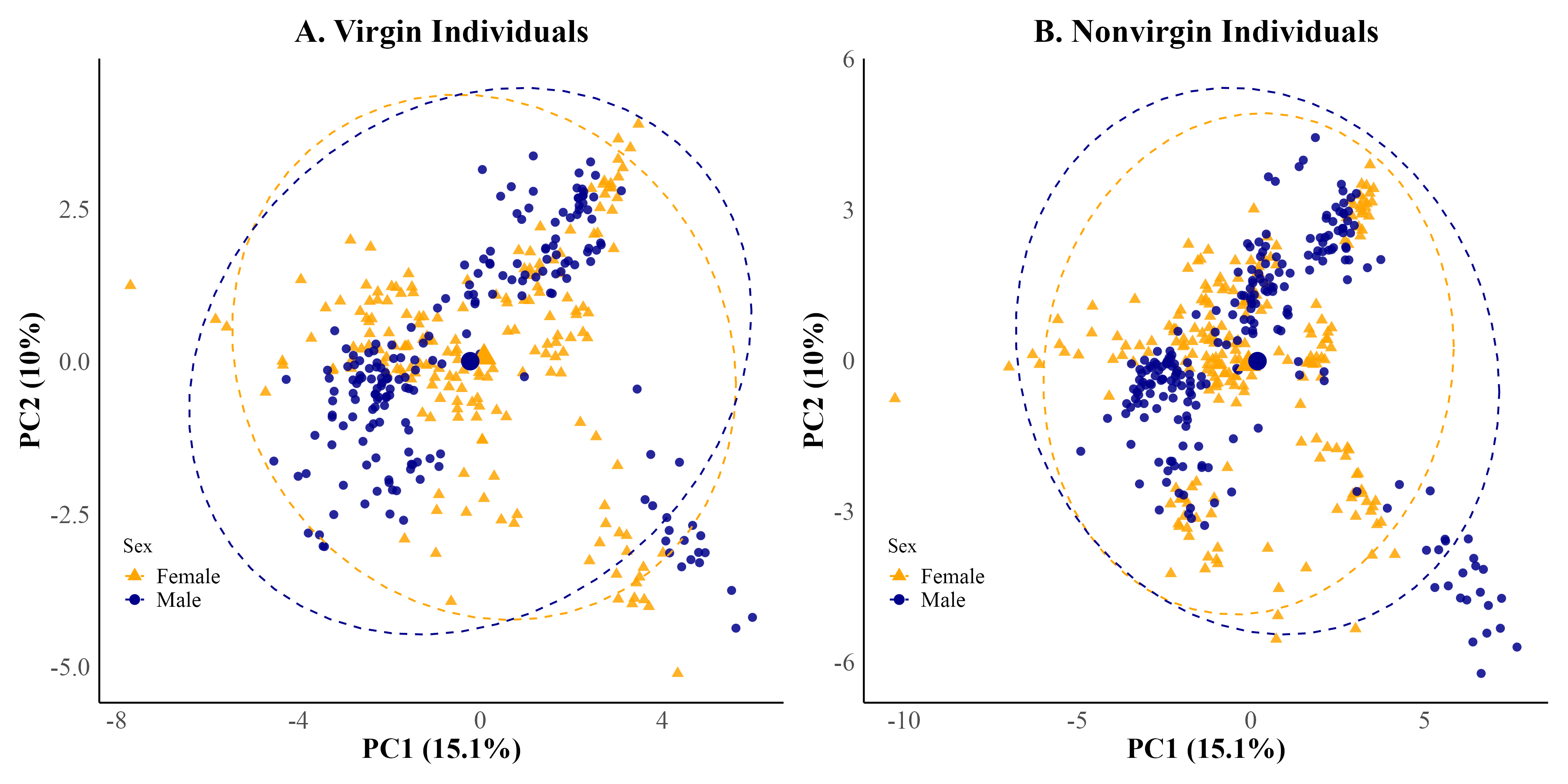

Supplement: Supplementary file 3 — Supplementary Material 3 [file 41598_2025_17544_MOESM3_ESM.tiff]
