# Supplementary material for: Cuticular hydrocarbon profiles in plump bush crickets vary according to species, sex and mating status
Source: Sci Rep. 2025 Sep 26;15:33233. doi: 10.1038/s41598-025-17544-7 (PMC12475074; doi:10.1038/s41598-025-17544-7)
Supplement: Supplementary file 4 — Supplementary Material 4 [file 41598_2025_17544_MOESM4_ESM.docx]

**Supplementary Table S1.** Mean relative abundance (%) of each CHC class in *Isophya* species, grouped by species, species group, sex, and mating status. Values represent the average percentage contribution of each CHC class to the total cuticular hydrocarbon (CHC) profile per individual. Standard deviations and sample sizes are also provided.

| **Species** | **Species Group** | **Sex** | **Mating Status** | **CHC_class** | **mean percentages** | **standard deviations** | **n** |
| --- | --- | --- | --- | --- | --- | --- | --- |
| *zernovi* | *zernovi* | F | NV | Alkadiene | 1.94 | 0.64 | 11 |
| *zernovi* | *zernovi* | F | NV | Alkene | 3.52 | 2.53 | 81 |
| *zernovi* | *zernovi* | F | NV | Methyl-branched alkane (dimethly) | 5.63 | 3.01 | 24 |
| *zernovi* | *zernovi* | F | NV | Methyl-branched alkane (monomethyl) | 2.44 | 0.83 | 20 |
| *zernovi* | *zernovi* | F | NV | Unknown | 2.45 | 0.96 | 28 |
| *zernovi* | *zernovi* | F | NV | n-Alkane | 6.53 | 6.64 | 282 |
| *zernovi* | *zernovi* | F | V | Alkadiene | 1.18 |  | 1 |
| *zernovi* | *zernovi* | F | V | Alkene | 4.02 | 3.05 | 62 |
| *zernovi* | *zernovi* | F | V | Methyl-branched alkane (dimethly) | 4.44 | 2.11 | 34 |
| *zernovi* | *zernovi* | F | V | Methyl-branched alkane (monomethyl) | 1.65 |  | 1 |
| *zernovi* | *zernovi* | F | V | Unknown | 2.62 | 0.88 | 38 |
| *zernovi* | *zernovi* | F | V | n-Alkane | 7.59 | 7.77 | 263 |
| *zernovi* | *zernovi* | M | NV | Alkadiene | 1.87 |  | 1 |
| *zernovi* | *zernovi* | M | NV | Alkene | 1.96 | 0.59 | 27 |
| *zernovi* | *zernovi* | M | NV | Methyl-branched alkane (monomethyl) | 5.10 | 3.45 | 99 |
| *zernovi* | *zernovi* | M | NV | Unknown | 2.54 | 1.60 | 26 |
| *zernovi* | *zernovi* | M | NV | n-Alkane | 5.58 | 5.38 | 336 |
| *zernovi* | *zernovi* | M | V | Alkadiene | 1.38 |  | 1 |
| *zernovi* | *zernovi* | M | V | Alkene | 4.25 | 2.81 | 14 |
| *zernovi* | *zernovi* | M | V | Methyl-branched alkane (monomethyl) | 4.73 | 3.36 | 98 |
| *zernovi* | *zernovi* | M | V | Unknown | 2.91 | 2.05 | 27 |
| *zernovi* | *zernovi* | M | V | n-Alkane | 6.32 | 6.96 | 300 |
| *autumnalis* | *zernovi* | F | NV | Alkene | 2.46 | 0.52 | 6 |
| *autumnalis* | *zernovi* | F | NV | Methyl-branched alkane (dimethly) | 4.31 | 2.22 | 22 |
| *autumnalis* | *zernovi* | F | NV | Methyl-branched alkane (monomethyl) | 2.50 | 1.07 | 18 |
| *autumnalis* | *zernovi* | F | NV | Unknown | 2.81 | 3.46 | 30 |
| *autumnalis* | *zernovi* | F | NV | n-Alkane | 6.93 | 6.81 | 247 |
| *autumnalis* | *zernovi* | F | V | Methyl-branched alkane (dimethly) | 4.00 | 2.20 | 44 |
| *autumnalis* | *zernovi* | F | V | Methyl-branched alkane (monomethyl) | 2.81 | 1.16 | 27 |
| *autumnalis* | *zernovi* | F | V | Unknown | 3.41 | 1.04 | 14 |
| *autumnalis* | *zernovi* | F | V | n-Alkane | 7.49 | 8.04 | 294 |
| *autumnalis* | *zernovi* | M | NV | Alkadiene | 1.16 | 0.10 | 2 |
| *autumnalis* | *zernovi* | M | NV | Alkene | 3.48 | 3.32 | 4 |
| *autumnalis* | *zernovi* | M | NV | Methyl-branched alkane (dimethly) | 3.49 | 2.16 | 23 |
| *autumnalis* | *zernovi* | M | NV | Methyl-branched alkane (monomethyl) | 5.55 | 3.48 | 68 |
| *autumnalis* | *zernovi* | M | NV | Unknown | 2.37 | 1.69 | 24 |
| *autumnalis* | *zernovi* | M | NV | n-Alkane | 6.21 | 5.47 | 277 |
| *autumnalis* | *zernovi* | M | V | Alkadiene | 1.51 |  | 1 |
| *autumnalis* | *zernovi* | M | V | Methyl-branched alkane (dimethly) | 3.75 | 2.06 | 52 |
| *autumnalis* | *zernovi* | M | V | Methyl-branched alkane (monomethyl) | 5.51 | 2.93 | 81 |
| *autumnalis* | *zernovi* | M | V | Unknown | 1.94 | 0.63 | 25 |
| *autumnalis* | *zernovi* | M | V | n-Alkane | 7.24 | 5.75 | 250 |
| *bicarinata* | *zernovi* | F | NV | Alkadiene | 3.42 |  | 1 |
| *bicarinata* | *zernovi* | F | NV | Alkene | 16.36 | 12.05 | 44 |
| *bicarinata* | *zernovi* | F | NV | Methyl-branched alkane (dimethly) | 3.22 | 1.76 | 29 |
| *bicarinata* | *zernovi* | F | NV | Methyl-branched alkane (monomethyl) | 2.24 | 1.14 | 7 |
| *bicarinata* | *zernovi* | F | NV | Unknown | 2.47 | 1.16 | 39 |
| *bicarinata* | *zernovi* | F | NV | n-Alkane | 5.83 | 6.28 | 261 |
| *bicarinata* | *zernovi* | F | V | Alkadiene | 2.36 | 1.63 | 2 |
| *bicarinata* | *zernovi* | F | V | Alkene | 15.43 | 12.85 | 66 |
| *bicarinata* | *zernovi* | F | V | Methyl-branched alkane (dimethly) | 2.99 | 1.40 | 19 |
| *bicarinata* | *zernovi* | F | V | Methyl-branched alkane (monomethyl) | 3.44 | 1.73 | 5 |
| *bicarinata* | *zernovi* | F | V | Unknown | 2.09 | 1.04 | 19 |
| *bicarinata* | *zernovi* | F | V | n-Alkane | 6.22 | 5.90 | 219 |
| *bicarinata* | *zernovi* | M | NV | Alkadiene | 1.64 | 0.40 | 5 |
| *bicarinata* | *zernovi* | M | NV | Alkene | 2.95 | 2.17 | 72 |
| *bicarinata* | *zernovi* | M | NV | Methyl-branched alkane (dimethly) | 2.45 | 0.67 | 28 |
| *bicarinata* | *zernovi* | M | NV | Methyl-branched alkane (monomethyl) | 4.70 | 3.71 | 96 |
| *bicarinata* | *zernovi* | M | NV | Unknown | 2.98 | 1.15 | 25 |
| *bicarinata* | *zernovi* | M | NV | n-Alkane | 5.14 | 5.24 | 318 |
| *bicarinata* | *zernovi* | M | V | Alkene | 3.96 | 2.70 | 49 |
| *bicarinata* | *zernovi* | M | V | Methyl-branched alkane (dimethly) | 2.16 | 0.63 | 11 |
| *bicarinata* | *zernovi* | M | V | Methyl-branched alkane (monomethyl) | 4.80 | 3.68 | 93 |
| *bicarinata* | *zernovi* | M | V | Unknown | 3.76 | 1.65 | 26 |
| *bicarinata* | *zernovi* | M | V | n-Alkane | 5.43 | 5.38 | 320 |
| *karadenizensis* | *zernovi* | F | NV | Alkene | 5.50 | 4.52 | 58 |
| *karadenizensis* | *zernovi* | F | NV | Methyl-branched alkane (monomethyl) | 1.94 | 0.66 | 3 |
| *karadenizensis* | *zernovi* | F | NV | Unknown | 2.23 | 0.81 | 15 |
| *karadenizensis* | *zernovi* | F | NV | n-Alkane | 7.84 | 8.29 | 256 |
| *karadenizensis* | *zernovi* | F | V | Alkene | 1.43 |  | 1 |
| *karadenizensis* | *zernovi* | F | V | Methyl-branched alkane (dimethly) | 2.88 | 1.74 | 5 |
| *karadenizensis* | *zernovi* | F | V | Unknown | 2.80 | 1.05 | 15 |
| *karadenizensis* | *zernovi* | F | V | n-Alkane | 7.71 | 7.28 | 252 |
| *karadenizensis* | *zernovi* | M | NV | Alkadiene | 3.75 |  | 1 |
| *karadenizensis* | *zernovi* | M | NV | Alkene | 3.31 | 2.37 | 106 |
| *karadenizensis* | *zernovi* | M | NV | Methyl-branched alkane (monomethyl) | 4.82 | 2.23 | 62 |
| *karadenizensis* | *zernovi* | M | NV | Unknown | 1.82 | 0.58 | 23 |
| *karadenizensis* | *zernovi* | M | NV | n-Alkane | 6.46 | 6.00 | 295 |
| *karadenizensis* | *zernovi* | M | V | Alkene | 3.58 | 3.11 | 110 |
| *karadenizensis* | *zernovi* | M | V | Methyl-branched alkane (monomethyl) | 5.15 | 2.64 | 66 |
| *karadenizensis* | *zernovi* | M | V | Unknown | 2.22 | 0.86 | 25 |
| *karadenizensis* | *zernovi* | M | V | n-Alkane | 6.20 | 5.60 | 276 |
| *karadenizensis* | *zernovi* | F | NV | Alkadiene | 3.49 | 1.33 | 10 |
| *nervosa* | *rectipennis* | F | NV | Alkadiene | 1.52 | 0.43 | 3 |
| *nervosa* | *rectipennis* | F | NV | Alkene | 3.53 | 1.96 | 42 |
| *nervosa* | *rectipennis* | F | NV | Methyl-branched alkane (dimethly) | 1.94 | 0.80 | 18 |
| *nervosa* | *rectipennis* | F | NV | Methyl-branched alkane (monomethyl) | 14.80 | 9.30 | 31 |
| *nervosa* | *rectipennis* | F | NV | n-Alkane | 6.48 | 5.50 | 209 |
| *nervosa* | *rectipennis* | F | V | Alkene | 2.88 | 1.76 | 57 |
| *nervosa* | *rectipennis* | F | V | Methyl-branched alkane (dimethly) | 1.73 | 0.39 | 21 |
| *nervosa* | *rectipennis* | F | V | Methyl-branched alkane (monomethyl) | 16.72 | 11.47 | 37 |
| *nervosa* | *rectipennis* | F | V | n-Alkane | 7.00 | 5.62 | 226 |
| *nervosa* | *rectipennis* | M | NV | Alkene | 1.73 | 0.63 | 19 |
| *nervosa* | *rectipennis* | M | NV | Methyl-branched alkane (monomethyl) | 6.57 | 8.06 | 73 |
| *nervosa* | *rectipennis* | M | NV | n-Alkane | 5.72 | 4.01 | 260 |
| *nervosa* | *rectipennis* | M | V | Alkene | 2.20 | 1.05 | 30 |
| *nervosa* | *rectipennis* | M | V | Methyl-branched alkane (dimethly) | 1.15 |  | 1 |
| *nervosa* | *rectipennis* | M | V | Methyl-branched alkane (monomethyl) | 7.19 | 7.22 | 68 |
| *nervosa* | *rectipennis* | M | V | n-Alkane | 5.45 | 3.37 | 320 |
| *obenbergeri* | *rectipennis* | F | NV | Alkene | 2.43 | 1.12 | 17 |
| *obenbergeri* | *rectipennis* | F | NV | Methyl-branched alkane (monomethyl) | 1.74 | 0.74 | 59 |
| *obenbergeri* | *rectipennis* | F | NV | n-Alkane | 8.59 | 6.57 | 216 |
| *obenbergeri* | *rectipennis* | F | V | Alkene | 1.54 | 0.26 | 9 |
| *obenbergeri* | *rectipennis* | F | V | Methyl-branched alkane (monomethyl) | 1.89 | 0.73 | 78 |
| *obenbergeri* | *rectipennis* | F | V | n-Alkane | 8.44 | 6.37 | 277 |
| *obenbergeri* | *rectipennis* | M | NV | Alkene | 4.75 | 6.99 | 5 |
| *obenbergeri* | *rectipennis* | M | NV | Methyl-branched alkane (monomethyl) | 2.26 | 1.35 | 82 |
| *obenbergeri* | *rectipennis* | M | NV | n-Alkane | 5.93 | 5.77 | 302 |
| *obenbergeri* | *rectipennis* | M | V | Methyl-branched alkane (monomethyl) | 4.01 | 2.65 | 75 |
| *obenbergeri* | *rectipennis* | M | V | Unknown | 1.27 | 0.07 | 2 |
| *obenbergeri* | *rectipennis* | M | V | n-Alkane | 6.03 | 4.52 | 364 |
| *rectipennis* | *rectipennis* | F | NV | Alkadiene | 4.40 | 1.34 | 22 |
| *rectipennis* | *rectipennis* | F | NV | Alkene | 4.22 | 1.69 | 19 |
| *rectipennis* | *rectipennis* | F | NV | Methyl-branched alkane (monomethyl) | 3.15 | 1.46 | 29 |
| *rectipennis* | *rectipennis* | F | NV | n-Alkane | 7.34 | 7.24 | 236 |
| *rectipennis* | *rectipennis* | F | V | Alkadiene | 3.97 | 1.69 | 28 |
| *rectipennis* | *rectipennis* | F | V | Alkene | 1.53 |  | 1 |
| *rectipennis* | *rectipennis* | F | V | Methyl-branched alkane (monomethyl) | 3.83 | 2.46 | 42 |
| *rectipennis* | *rectipennis* | F | V | n-Alkane | 7.35 | 6.96 | 303 |
| *rectipennis* | *rectipennis* | M | NV | Alkadiene | 3.18 | 0.74 | 19 |
| *rectipennis* | *rectipennis* | M | NV | Alkene | 3.22 | 2.14 | 38 |
| *rectipennis* | *rectipennis* | M | NV | Methyl-branched alkane (monomethyl) | 2.79 | 1.46 | 55 |
| *rectipennis* | *rectipennis* | M | NV | Unknown | 1.09 |  | 1 |
| *rectipennis* | *rectipennis* | M | NV | n-Alkane | 6.42 | 6.31 | 259 |
| *rectipennis* | *rectipennis* | M | V | Alkadiene | 2.85 | 1.26 | 25 |
| *rectipennis* | *rectipennis* | M | V | Alkene | 1.49 | 0.33 | 4 |
| *rectipennis* | *rectipennis* | M | V | Methyl-branched alkane (dimethly) | 1.85 | 0.85 | 20 |
| *rectipennis* | *rectipennis* | M | V | Methyl-branched alkane (monomethyl) | 4.35 | 2.37 | 92 |
| *rectipennis* | *rectipennis* | M | V | n-Alkane | 5.35 | 5.30 | 371 |
|  |  |  |  |  |  |  |  |
|  |  |  |  |  |  |  |  |
|  |  |  |  |  |  |  |  |
|  |  |  |  |  |  |  |  |
|  |  |  |  |  |  |  |  |
|  |  |  |  |  |  |  |  |
|  |  |  |  |  |  |  |  |
| *stenocauda* | *rectipennis* | F | NV | Alkadiene | 1.81 | 0.34 | 8 |
| *stenocauda* | *rectipennis* | F | NV | Alkene | 2.43 | 0.91 | 92 |
| *stenocauda* | *rectipennis* | F | NV | Methyl-branched alkane (monomethyl) | 1.77 | 0.91 | 16 |
| *stenocauda* | *rectipennis* | F | NV | n-Alkane | 7.92 | 6.26 | 219 |
| *stenocauda* | *rectipennis* | F | V | Alkadiene | 2.69 | 1.29 | 73 |
| *stenocauda* | *rectipennis* | F | V | Alkene | 2.85 | 1.44 | 85 |
| *stenocauda* | *rectipennis* | F | V | Methyl-branched alkane (monomethyl) | 1.63 | 0.54 | 12 |
| *stenocauda* | *rectipennis* | F | V | n-Alkane | 7.07 | 7.17 | 289 |
| *stenocauda* | *rectipennis* | M | NV | Alkadiene | 2.66 | 0.82 | 4 |
| *stenocauda* | *rectipennis* | M | NV | Alkene | 4.36 | 1.87 | 41 |
| *stenocauda* | *rectipennis* | M | NV | Methyl-branched alkane (monomethyl) | 1.94 | 0.65 | 89 |
| *stenocauda* | *rectipennis* | M | NV | Unknown | 1.92 | 0.54 | 5 |
| *stenocauda* | *rectipennis* | M | NV | n-Alkane | 5.84 | 4.90 | 279 |
| *stenocauda* | *rectipennis* | M | V | Alkene | 3.36 | 2.22 | 77 |
| *stenocauda* | *rectipennis* | M | V | Methyl-branched alkane (monomethyl) | 1.95 | 0.79 | 75 |
| *stenocauda* | *rectipennis* | M | V | n-Alkane | 4.98 | 4.31 | 401 |
| *ilkazi* | *rectipennis* | F | NV | Alkadiene | 1.83 |  | 1 |
| *ilkazi* | *rectipennis* | F | NV | Alkene | 4.35 | 3.09 | 21 |
| *ilkazi* | *rectipennis* | F | NV | Methyl-branched alkane (monomethyl) | 2.21 | 0.67 | 22 |
| *ilkazi* | *rectipennis* | F | NV | n-Alkane | 6.76 | 7.00 | 186 |
| *ilkazi* | *rectipennis* | F | V | Alkene | 1.56 | 0.27 | 15 |
| *ilkazi* | *rectipennis* | F | V | Methyl-branched alkane (monomethyl) | 4.27 | 2.50 | 46 |
| *ilkazi* | *rectipennis* | F | V | Unknown | 1.75 | 0.49 | 5 |
| *ilkazi* | *rectipennis* | F | V | n-Alkane | 6.13 | 8.44 | 338 |
| *ilkazi* | *rectipennis* | M | NV | Alkadiene | 1.77 | 0.51 | 19 |
| *ilkazi* | *rectipennis* | M | NV | Alkene | 5.80 | 2.98 | 18 |
| *ilkazi* | *rectipennis* | M | NV | Methyl-branched alkane (monomethyl) | 2.39 | 0.95 | 38 |
| *ilkazi* | *rectipennis* | M | NV | n-Alkane | 6.01 | 5.16 | 195 |
| *ilkazi* | *rectipennis* | M | V | Alkadiene | 1.68 | 0.59 | 12 |
| *ilkazi* | *rectipennis* | M | V | Alkene | 5.05 | 3.27 | 38 |
| *ilkazi* | *rectipennis* | M | V | Methyl-branched alkane (monomethyl) | 3.37 | 1.61 | 69 |
| *ilkazi* | *rectipennis* | M | V | Unknown | 2.01 | 1.51 | 7 |
| *ilkazi* | *rectipennis* | M | V | n-Alkane | 5.26 | 4.71 | 388 |
| *staneki* | *staneki* | F | V | Methyl-branched alkane (monomethyl) | 2.64 | 1.57 | 19 |
| *staneki* | *staneki* | F | V | Unknown | 1.55 | 0.33 | 5 |
| *staneki* | *staneki* | F | V | n-Alkane | 5.97 | 6.40 | 74 |
| *staneki* | *staneki* | M | V | Alkadiene | 1.10 |  | 1 |
| *staneki* | *staneki* | M | V | Methyl-branched alkane (monomethyl) | 4.36 | 1.73 | 15 |
| *staneki* | *staneki* | M | V | Unknown | 1.73 | 0.40 | 10 |
| *staneki* | *staneki* | M | V | n-Alkane | 6.40 | 5.63 | 65 |
|  |  |  |  |  |  |  |  |
|  |  |  |  |  |  |  |  |
|  |  |  |  |  |  |  |  |
|  |  |  |  |  |  |  |  |
|  |  |  |  |  |  |  |  |
|  |  |  |  |  |  |  |  |
|  |  |  |  |  |  |  |  |
|  |  |  |  |  |  |  |  |
|  |  |  |  |  |  |  |  |
|  |  |  |  |  |  |  |  |
|  |  |  |  |  |  |  |  |
|  |  |  |  |  |  |  |  |
|  |  |  |  |  |  |  |  |
|  |  |  |  |  |  |  |  |
|  |  |  |  |  |  |  |  |
