# Supplementary material for: Cuticular hydrocarbon profiles in plump bush crickets vary according to species, sex and mating status
Source: Sci Rep. 2025 Sep 26;15:33233. doi: 10.1038/s41598-025-17544-7 (PMC12475074; doi:10.1038/s41598-025-17544-7)
Supplement: Supplementary file 5 — Supplementary Material 5 [file 41598_2025_17544_MOESM5_ESM.docx]

**Supp. Table 2.** Results of pairwise species comparisons within each combination of sex and mating status across PC1 to PC5. Pairwise comparisons were performed using estimated marginal means (emmeans) based on ANOVA models including species, sex, mating status, and all interactions. P-values were adjusted using the Holm method to control for family-wise error rate.

| **Sex** | **MS** | **contrast** | **PC** | ***p*** |
| --- | --- | --- | --- | --- |
| F | NV | *autumnalis - bicarinata* | PC1 | 0.027 |
| F | NV | *autumnalis - ilkazi* | PC1 | <0.001 |
| F | NV | *autumnalis - karadenizensis* | PC1 | <0.001 |
| F | NV | *autumnalis - nervosa* | PC1 | <0.001 |
| F | NV | *autumnalis - obenbergeri* | PC1 | <0.001 |
| F | NV | *autumnalis - rectipennis* | PC1 | <0.001 |
| F | NV | *autumnalis - stenocauda* | PC1 | <0.001 |
| F | NV | *autumnalis - zernovi* | PC1 | 0.586 |
| F | NV | *bicarinata - ilkazi* | PC1 | <0.001 |
| F | NV | *bicarinata - karadenizensis* | PC1 | <0.001 |
| F | NV | *bicarinata - nervosa* | PC1 | <0.001 |
| F | NV | *bicarinata - obenbergeri* | PC1 | <0.001 |
| F | NV | *bicarinata - rectipennis* | PC1 | <0.001 |
| F | NV | *bicarinata - stenocauda* | PC1 | <0.001 |
| F | NV | *bicarinata - zernovi* | PC1 | 0.224 |
| F | NV | *ilkazi - karadenizensis* | PC1 | 0.443 |
| F | NV | *ilkazi - nervosa* | PC1 | <0.001 |
| F | NV | *ilkazi - obenbergeri* | PC1 | <0.001 |
| F | NV | *ilkazi - rectipennis* | PC1 | <0.001 |
| F | NV | *ilkazi - stenocauda* | PC1 | <0.001 |
| F | NV | *ilkazi - zernovi* | PC1 | <0.001 |
| F | NV | *karadenizensis - nervosa* | PC1 | <0.001 |
| F | NV | *karadenizensis - obenbergeri* | PC1 | <0.001 |
| F | NV | *karadenizensis - rectipennis* | PC1 | <0.001 |
| F | NV | *karadenizensis - stenocauda* | PC1 | <0.001 |
| F | NV | *karadenizensis - zernovi* | PC1 | <0.001 |
| F | NV | *nervosa - obenbergeri* | PC1 | <0.001 |
| F | NV | *nervosa - rectipennis* | PC1 | 0.033 |
| F | NV | *nervosa - stenocauda* | PC1 | <0.001 |
| F | NV | *nervosa - zernovi* | PC1 | <0.001 |
| F | NV | *obenbergeri - rectipennis* | PC1 | <0.001 |
| F | NV | *obenbergeri - stenocauda* | PC1 | 0.595 |
| F | NV | *obenbergeri - zernovi* | PC1 | <0.001 |
| F | NV | *rectipennis - stenocauda* | PC1 | <0.001 |
| F | NV | *rectipennis - zernovi* | PC1 | <0.001 |
| F | NV | *stenocauda - zernovi* | PC1 | <0.001 |
| M | NV | *autumnalis - bicarinata* | PC1 | <0.001 |
| M | NV | *autumnalis - ilkazi* | PC1 | <0.001 |
| M | NV | *autumnalis - karadenizensis* | PC1 | 0.066 |
| M | NV | *autumnalis - nervosa* | PC1 | <0.001 |
| M | NV | *autumnalis - obenbergeri* | PC1 | <0.001 |
| M | NV | *autumnalis - rectipennis* | PC1 | <0.001 |
| M | NV | *autumnalis - stenocauda* | PC1 | <0.001 |
| M | NV | *autumnalis - zernovi* | PC1 | <0.001 |
| M | NV | *bicarinata - ilkazi* | PC1 | <0.001 |
| M | NV | *bicarinata - karadenizensis* | PC1 | 0.024 |
| M | NV | *bicarinata - nervosa* | PC1 | <0.001 |
| M | NV | *bicarinata - obenbergeri* | PC1 | <0.001 |
| M | NV | *bicarinata - rectipennis* | PC1 | <0.001 |
| M | NV | *bicarinata - stenocauda* | PC1 | <0.001 |
| M | NV | *bicarinata - zernovi* | PC1 | 0.788 |
| M | NV | *ilkazi - karadenizensis* | PC1 | <0.001 |
| M | NV | *ilkazi - nervosa* | PC1 | <0.001 |
| M | NV | *ilkazi - obenbergeri* | PC1 | <0.001 |
| M | NV | *ilkazi - rectipennis* | PC1 | <0.001 |
| M | NV | *ilkazi - stenocauda* | PC1 | 0.637 |
| M | NV | *ilkazi - zernovi* | PC1 | <0.001 |
| M | NV | *karadenizensis - nervosa* | PC1 | <0.001 |
| M | NV | *karadenizensis - obenbergeri* | PC1 | <0.001 |
| M | NV | *karadenizensis - rectipennis* | PC1 | <0.001 |
| M | NV | *karadenizensis - stenocauda* | PC1 | <0.001 |
| M | NV | *karadenizensis - zernovi* | PC1 | 0.053 |
| M | NV | *nervosa - obenbergeri* | PC1 | 0.007 |
| M | NV | *nervosa - rectipennis* | PC1 | <0.001 |
| M | NV | *nervosa - stenocauda* | PC1 | <0.001 |
| M | NV | *nervosa - zernovi* | PC1 | <0.001 |
| M | NV | *obenbergeri - rectipennis* | PC1 | <0.001 |
| M | NV | *obenbergeri - stenocauda* | PC1 | <0.001 |
| M | NV | *obenbergeri - zernovi* | PC1 | <0.001 |
| M | NV | *rectipennis - stenocauda* | PC1 | <0.001 |
| M | NV | *rectipennis - zernovi* | PC1 | <0.001 |
| M | NV | *stenocauda - zernovi* | PC1 | <0.001 |
| F | V | *autumnalis - bicarinata* | PC1 | <0.001 |
| F | V | *autumnalis - ilkazi* | PC1 | <0.001 |
| F | V | *autumnalis - karadenizensis* | PC1 | <0.001 |
| F | V | *autumnalis - nervosa* | PC1 | <0.001 |
| F | V | *autumnalis - obenbergeri* | PC1 | <0.001 |
| F | V | *autumnalis - rectipennis* | PC1 | <0.001 |
| F | V | *autumnalis - staneki* | PC1 | <0.001 |
| F | V | *autumnalis - stenocauda* | PC1 | <0.001 |
| F | V | *autumnalis - zernovi* | PC1 | <0.001 |
| F | V | *bicarinata - ilkazi* | PC1 | 0.058 |
| F | V | *bicarinata - karadenizensis* | PC1 | 0.141 |
| F | V | *bicarinata - nervosa* | PC1 | <0.001 |
| F | V | *bicarinata - obenbergeri* | PC1 | <0.001 |
| F | V | *bicarinata - rectipennis* | PC1 | <0.001 |
| F | V | *bicarinata - staneki* | PC1 | <0.001 |
| F | V | *bicarinata - stenocauda* | PC1 | <0.001 |
| F | V | *bicarinata - zernovi* | PC1 | 0.002 |
| F | V | *ilkazi - karadenizensis* | PC1 | 0.769 |
| F | V | *ilkazi - nervosa* | PC1 | <0.001 |
| F | V | *ilkazi - obenbergeri* | PC1 | <0.001 |
| F | V | *ilkazi - rectipennis* | PC1 | <0.001 |
| F | V | *ilkazi - staneki* | PC1 | 0.001 |
| F | V | *ilkazi - stenocauda* | PC1 | <0.001 |
| F | V | *ilkazi - zernovi* | PC1 | <0.001 |
| F | V | *karadenizensis - nervosa* | PC1 | <0.001 |
| F | V | *karadenizensis - obenbergeri* | PC1 | <0.001 |
| F | V | *karadenizensis - rectipennis* | PC1 | <0.001 |
| F | V | *karadenizensis - staneki* | PC1 | <0.001 |
| F | V | *karadenizensis - stenocauda* | PC1 | <0.001 |
| F | V | *karadenizensis - zernovi* | PC1 | <0.001 |
| F | V | *nervosa - obenbergeri* | PC1 | <0.001 |
| F | V | *nervosa - rectipennis* | PC1 | 0.348 |
| F | V | *nervosa - staneki* | PC1 | <0.001 |
| F | V | *nervosa - stenocauda* | PC1 | <0.001 |
| F | V | *nervosa - zernovi* | PC1 | <0.001 |
| F | V | *obenbergeri - rectipennis* | PC1 | <0.001 |
| F | V | *obenbergeri - staneki* | PC1 | 0.348 |
| F | V | *obenbergeri - stenocauda* | PC1 | <0.001 |
| F | V | *obenbergeri - zernovi* | PC1 | <0.001 |
| F | V | *rectipennis - staneki* | PC1 | <0.001 |
| F | V | *rectipennis - stenocauda* | PC1 | <0.001 |
| F | V | *rectipennis - zernovi* | PC1 | <0.001 |
| F | V | *staneki - stenocauda* | PC1 | 0.615 |
| F | V | *staneki - zernovi* | PC1 | <0.001 |
| F | V | *stenocauda - zernovi* | PC1 | <0.001 |
| M | V | *autumnalis - bicarinata* | PC1 | <0.001 |
| M | V | *autumnalis - ilkazi* | PC1 | <0.001 |
| M | V | *autumnalis - karadenizensis* | PC1 | 0.527 |
| M | V | *autumnalis - nervosa* | PC1 | <0.001 |
| M | V | *autumnalis - obenbergeri* | PC1 | <0.001 |
| M | V | *autumnalis - rectipennis* | PC1 | <0.001 |
| M | V | *autumnalis - staneki* | PC1 | <0.001 |
| M | V | *autumnalis - stenocauda* | PC1 | <0.001 |
| M | V | *autumnalis - zernovi* | PC1 | 0.001 |
| M | V | *bicarinata - ilkazi* | PC1 | <0.001 |
| M | V | *bicarinata - karadenizensis* | PC1 | <0.001 |
| M | V | *bicarinata - nervosa* | PC1 | <0.001 |
| M | V | *bicarinata - obenbergeri* | PC1 | <0.001 |
| M | V | *bicarinata - rectipennis* | PC1 | <0.001 |
| M | V | *bicarinata - staneki* | PC1 | <0.001 |
| M | V | *bicarinata - stenocauda* | PC1 | <0.001 |
| M | V | *bicarinata - zernovi* | PC1 | 0.527 |
| M | V | *ilkazi - karadenizensis* | PC1 | <0.001 |
| M | V | *ilkazi - nervosa* | PC1 | <0.001 |
| M | V | *ilkazi - obenbergeri* | PC1 | <0.001 |
| M | V | *ilkazi - rectipennis* | PC1 | <0.001 |
| M | V | *ilkazi - staneki* | PC1 | 0.014 |
| M | V | *ilkazi - stenocauda* | PC1 | 0.527 |
| M | V | *ilkazi - zernovi* | PC1 | <0.001 |
| M | V | *karadenizensis - nervosa* | PC1 | <0.001 |
| M | V | *karadenizensis - obenbergeri* | PC1 | <0.001 |
| M | V | *karadenizensis - rectipennis* | PC1 | <0.001 |
| M | V | *karadenizensis - staneki* | PC1 | <0.001 |
| M | V | *karadenizensis - stenocauda* | PC1 | <0.001 |
| M | V | *karadenizensis - zernovi* | PC1 | <0.001 |
| M | V | *nervosa - obenbergeri* | PC1 | <0.001 |
| M | V | *nervosa - rectipennis* | PC1 | <0.001 |
| M | V | *nervosa - staneki* | PC1 | <0.001 |
| M | V | *nervosa - stenocauda* | PC1 | <0.001 |
| M | V | *nervosa - zernovi* | PC1 | <0.001 |
| M | V | *obenbergeri - rectipennis* | PC1 | <0.001 |
| M | V | *obenbergeri - staneki* | PC1 | 0.527 |
| M | V | *obenbergeri - stenocauda* | PC1 | <0.001 |
| M | V | *obenbergeri - zernovi* | PC1 | <0.001 |
| M | V | *rectipennis - staneki* | PC1 | <0.001 |
| M | V | *rectipennis - stenocauda* | PC1 | <0.001 |
| M | V | *rectipennis - zernovi* | PC1 | <0.001 |
| M | V | *staneki - stenocauda* | PC1 | 0.15 |
| M | V | *staneki - zernovi* | PC1 | <0.001 |
| M | V | *stenocauda - zernovi* | PC1 | <0.001 |
| F | NV | *autumnalis - bicarinata* | PC2 | 0.139 |
| F | NV | *autumnalis - ilkazi* | PC2 | <0.001 |
| F | NV | *autumnalis - karadenizensis* | PC2 | 0.139 |
| F | NV | *autumnalis - nervosa* | PC2 | <0.001 |
| F | NV | *autumnalis - obenbergeri* | PC2 | 0.018 |
| F | NV | *autumnalis - rectipennis* | PC2 | <0.001 |
| F | NV | *autumnalis - stenocauda* | PC2 | <0.001 |
| F | NV | *autumnalis - zernovi* | PC2 | <0.001 |
| F | NV | *bicarinata - ilkazi* | PC2 | <0.001 |
| F | NV | *bicarinata - karadenizensis* | PC2 | 0.927 |
| F | NV | *bicarinata - nervosa* | PC2 | <0.001 |
| F | NV | *bicarinata - obenbergeri* | PC2 | <0.001 |
| F | NV | *bicarinata - rectipennis* | PC2 | <0.001 |
| F | NV | *bicarinata - stenocauda* | PC2 | <0.001 |
| F | NV | *bicarinata - zernovi* | PC2 | <0.001 |
| F | NV | *ilkazi - karadenizensis* | PC2 | <0.001 |
| F | NV | *ilkazi - nervosa* | PC2 | <0.001 |
| F | NV | *ilkazi - obenbergeri* | PC2 | <0.001 |
| F | NV | *ilkazi - rectipennis* | PC2 | <0.001 |
| F | NV | *ilkazi - stenocauda* | PC2 | <0.001 |
| F | NV | *ilkazi - zernovi* | PC2 | <0.001 |
| F | NV | *karadenizensis - nervosa* | PC2 | <0.001 |
| F | NV | *karadenizensis - obenbergeri* | PC2 | <0.001 |
| F | NV | *karadenizensis - rectipennis* | PC2 | <0.001 |
| F | NV | *karadenizensis - stenocauda* | PC2 | <0.001 |
| F | NV | *karadenizensis - zernovi* | PC2 | <0.001 |
| F | NV | *nervosa - obenbergeri* | PC2 | <0.001 |
| F | NV | *nervosa - rectipennis* | PC2 | <0.001 |
| F | NV | *nervosa - stenocauda* | PC2 | <0.001 |
| F | NV | *nervosa - zernovi* | PC2 | <0.001 |
| F | NV | *obenbergeri - rectipennis* | PC2 | <0.001 |
| F | NV | *obenbergeri - stenocauda* | PC2 | <0.001 |
| F | NV | *obenbergeri - zernovi* | PC2 | 0.159 |
| F | NV | *rectipennis - stenocauda* | PC2 | <0.001 |
| F | NV | *rectipennis - zernovi* | PC2 | <0.001 |
| F | NV | *stenocauda - zernovi* | PC2 | <0.001 |
| M | NV | *autumnalis - bicarinata* | PC2 | <0.001 |
| M | NV | *autumnalis - ilkazi* | PC2 | <0.001 |
| M | NV | *autumnalis - karadenizensis* | PC2 | 1 |
| M | NV | *autumnalis - nervosa* | PC2 | <0.001 |
| M | NV | *autumnalis - obenbergeri* | PC2 | 1 |
| M | NV | *autumnalis - rectipennis* | PC2 | <0.001 |
| M | NV | *autumnalis - stenocauda* | PC2 | <0.001 |
| M | NV | *autumnalis - zernovi* | PC2 | 1 |
| M | NV | *bicarinata - ilkazi* | PC2 | 1 |
| M | NV | *bicarinata - karadenizensis* | PC2 | <0.001 |
| M | NV | *bicarinata - nervosa* | PC2 | <0.001 |
| M | NV | *bicarinata - obenbergeri* | PC2 | <0.001 |
| M | NV | *bicarinata - rectipennis* | PC2 | <0.001 |
| M | NV | *bicarinata - stenocauda* | PC2 | <0.001 |
| M | NV | *bicarinata - zernovi* | PC2 | <0.001 |
| M | NV | *ilkazi - karadenizensis* | PC2 | <0.001 |
| M | NV | *ilkazi - nervosa* | PC2 | <0.001 |
| M | NV | *ilkazi - obenbergeri* | PC2 | <0.001 |
| M | NV | *ilkazi - rectipennis* | PC2 | <0.001 |
| M | NV | *ilkazi - stenocauda* | PC2 | <0.001 |
| M | NV | *ilkazi - zernovi* | PC2 | <0.001 |
| M | NV | *karadenizensis - nervosa* | PC2 | <0.001 |
| M | NV | *karadenizensis - obenbergeri* | PC2 | 1 |
| M | NV | *karadenizensis - rectipennis* | PC2 | <0.001 |
| M | NV | *karadenizensis - stenocauda* | PC2 | <0.001 |
| M | NV | *karadenizensis - zernovi* | PC2 | 1 |
| M | NV | *nervosa - obenbergeri* | PC2 | <0.001 |
| M | NV | *nervosa - rectipennis* | PC2 | <0.001 |
| M | NV | *nervosa - stenocauda* | PC2 | <0.001 |
| M | NV | *nervosa - zernovi* | PC2 | <0.001 |
| M | NV | *obenbergeri - rectipennis* | PC2 | <0.001 |
| M | NV | *obenbergeri - stenocauda* | PC2 | <0.001 |
| M | NV | *obenbergeri - zernovi* | PC2 | 1 |
| M | NV | *rectipennis - stenocauda* | PC2 | <0.001 |
| M | NV | *rectipennis - zernovi* | PC2 | <0.001 |
| M | NV | *stenocauda - zernovi* | PC2 | <0.001 |
| F | V | *autumnalis - bicarinata* | PC2 | 0.515 |
| F | V | *autumnalis - ilkazi* | PC2 | <0.001 |
| F | V | *autumnalis - karadenizensis* | PC2 | 0.826 |
| F | V | *autumnalis - nervosa* | PC2 | <0.001 |
| F | V | *autumnalis - obenbergeri* | PC2 | <0.001 |
| F | V | *autumnalis - rectipennis* | PC2 | <0.001 |
| F | V | *autumnalis - staneki* | PC2 | <0.001 |
| F | V | *autumnalis - stenocauda* | PC2 | <0.001 |
| F | V | *autumnalis - zernovi* | PC2 | <0.001 |
| F | V | *bicarinata - ilkazi* | PC2 | <0.001 |
| F | V | *bicarinata - karadenizensis* | PC2 | 0.826 |
| F | V | *bicarinata - nervosa* | PC2 | <0.001 |
| F | V | *bicarinata - obenbergeri* | PC2 | 0.002 |
| F | V | *bicarinata - rectipennis* | PC2 | <0.001 |
| F | V | *bicarinata - staneki* | PC2 | <0.001 |
| F | V | *bicarinata - stenocauda* | PC2 | <0.001 |
| F | V | *bicarinata - zernovi* | PC2 | <0.001 |
| F | V | *ilkazi - karadenizensis* | PC2 | <0.001 |
| F | V | *ilkazi - nervosa* | PC2 | <0.001 |
| F | V | *ilkazi - obenbergeri* | PC2 | <0.001 |
| F | V | *ilkazi - rectipennis* | PC2 | <0.001 |
| F | V | *ilkazi - staneki* | PC2 | 0.006 |
| F | V | *ilkazi - stenocauda* | PC2 | <0.001 |
| F | V | *ilkazi - zernovi* | PC2 | <0.001 |
| F | V | *karadenizensis - nervosa* | PC2 | <0.001 |
| F | V | *karadenizensis - obenbergeri* | PC2 | <0.001 |
| F | V | *karadenizensis - rectipennis* | PC2 | <0.001 |
| F | V | *karadenizensis - staneki* | PC2 | <0.001 |
| F | V | *karadenizensis - stenocauda* | PC2 | <0.001 |
| F | V | *karadenizensis - zernovi* | PC2 | <0.001 |
| F | V | *nervosa - obenbergeri* | PC2 | <0.001 |
| F | V | *nervosa - rectipennis* | PC2 | <0.001 |
| F | V | *nervosa - staneki* | PC2 | <0.001 |
| F | V | *nervosa - stenocauda* | PC2 | <0.001 |
| F | V | *nervosa - zernovi* | PC2 | <0.001 |
| F | V | *obenbergeri - rectipennis* | PC2 | <0.001 |
| F | V | *obenbergeri - staneki* | PC2 | <0.001 |
| F | V | *obenbergeri - stenocauda* | PC2 | <0.001 |
| F | V | *obenbergeri - zernovi* | PC2 | 0.001 |
| F | V | *rectipennis - staneki* | PC2 | 0.62 |
| F | V | *rectipennis - stenocauda* | PC2 | <0.001 |
| F | V | *rectipennis - zernovi* | PC2 | <0.001 |
| F | V | *staneki - stenocauda* | PC2 | <0.001 |
| F | V | *staneki - zernovi* | PC2 | <0.001 |
| F | V | *stenocauda - zernovi* | PC2 | <0.001 |
| M | V | *autumnalis - bicarinata* | PC2 | <0.001 |
| M | V | *autumnalis - ilkazi* | PC2 | <0.001 |
| M | V | *autumnalis - karadenizensis* | PC2 | 0.722 |
| M | V | *autumnalis - nervosa* | PC2 | <0.001 |
| M | V | *autumnalis - obenbergeri* | PC2 | 0.476 |
| M | V | *autumnalis - rectipennis* | PC2 | <0.001 |
| M | V | *autumnalis - staneki* | PC2 | <0.001 |
| M | V | *autumnalis - stenocauda* | PC2 | <0.001 |
| M | V | *autumnalis - zernovi* | PC2 | 0.042 |
| M | V | *bicarinata - ilkazi* | PC2 | 0.722 |
| M | V | *bicarinata - karadenizensis* | PC2 | <0.001 |
| M | V | *bicarinata - nervosa* | PC2 | <0.001 |
| M | V | *bicarinata - obenbergeri* | PC2 | <0.001 |
| M | V | *bicarinata - rectipennis* | PC2 | <0.001 |
| M | V | *bicarinata - staneki* | PC2 | <0.001 |
| M | V | *bicarinata - stenocauda* | PC2 | <0.001 |
| M | V | *bicarinata - zernovi* | PC2 | <0.001 |
| M | V | *ilkazi - karadenizensis* | PC2 | <0.001 |
| M | V | *ilkazi - nervosa* | PC2 | <0.001 |
| M | V | *ilkazi - obenbergeri* | PC2 | <0.001 |
| M | V | *ilkazi - rectipennis* | PC2 | <0.001 |
| M | V | *ilkazi - staneki* | PC2 | 0.007 |
| M | V | *ilkazi - stenocauda* | PC2 | <0.001 |
| M | V | *ilkazi - zernovi* | PC2 | <0.001 |
| M | V | *karadenizensis - nervosa* | PC2 | <0.001 |
| M | V | *karadenizensis - obenbergeri* | PC2 | 0.722 |
| M | V | *karadenizensis - rectipennis* | PC2 | <0.001 |
| M | V | *karadenizensis - staneki* | PC2 | <0.001 |
| M | V | *karadenizensis - stenocauda* | PC2 | <0.001 |
| M | V | *karadenizensis - zernovi* | PC2 | 0.015 |
| M | V | *nervosa - obenbergeri* | PC2 | <0.001 |
| M | V | *nervosa - rectipennis* | PC2 | <0.001 |
| M | V | *nervosa - staneki* | PC2 | <0.001 |
| M | V | *nervosa - stenocauda* | PC2 | <0.001 |
| M | V | *nervosa - zernovi* | PC2 | <0.001 |
| M | V | *obenbergeri - rectipennis* | PC2 | <0.001 |
| M | V | *obenbergeri - staneki* | PC2 | <0.001 |
| M | V | *obenbergeri - stenocauda* | PC2 | <0.001 |
| M | V | *obenbergeri - zernovi* | PC2 | <0.001 |
| M | V | *rectipennis - staneki* | PC2 | <0.001 |
| M | V | *rectipennis - stenocauda* | PC2 | <0.001 |
| M | V | *rectipennis - zernovi* | PC2 | <0.001 |
| M | V | *staneki - stenocauda* | PC2 | <0.001 |
| M | V | *staneki - zernovi* | PC2 | <0.001 |
| M | V | *stenocauda - zernovi* | PC2 | <0.001 |
| F | NV | *autumnalis - bicarinata* | PC3 | 0.013 |
| F | NV | *autumnalis - ilkazi* | PC3 | <0.001 |
| F | NV | *autumnalis - karadenizensis* | PC3 | 1 |
| F | NV | *autumnalis - nervosa* | PC3 | <0.001 |
| F | NV | *autumnalis - obenbergeri* | PC3 | 0.001 |
| F | NV | *autumnalis - rectipennis* | PC3 | <0.001 |
| F | NV | *autumnalis - stenocauda* | PC3 | 1 |
| F | NV | *autumnalis - zernovi* | PC3 | 0.719 |
| F | NV | *bicarinata - ilkazi* | PC3 | <0.001 |
| F | NV | *bicarinata - karadenizensis* | PC3 | 0.002 |
| F | NV | *bicarinata - nervosa* | PC3 | 0.028 |
| F | NV | *bicarinata - obenbergeri* | PC3 | <0.001 |
| F | NV | *bicarinata - rectipennis* | PC3 | <0.001 |
| F | NV | *bicarinata - stenocauda* | PC3 | 0.037 |
| F | NV | *bicarinata - zernovi* | PC3 | 0.384 |
| F | NV | *ilkazi - karadenizensis* | PC3 | <0.001 |
| F | NV | *ilkazi - nervosa* | PC3 | <0.001 |
| F | NV | *ilkazi - obenbergeri* | PC3 | <0.001 |
| F | NV | *ilkazi - rectipennis* | PC3 | <0.001 |
| F | NV | *ilkazi - stenocauda* | PC3 | <0.001 |
| F | NV | *ilkazi - zernovi* | PC3 | <0.001 |
| F | NV | *karadenizensis - nervosa* | PC3 | <0.001 |
| F | NV | *karadenizensis - obenbergeri* | PC3 | 0.002 |
| F | NV | *karadenizensis - rectipennis* | PC3 | <0.001 |
| F | NV | *karadenizensis - stenocauda* | PC3 | 1 |
| F | NV | *karadenizensis - zernovi* | PC3 | 0.384 |
| F | NV | *nervosa - obenbergeri* | PC3 | <0.001 |
| F | NV | *nervosa - rectipennis* | PC3 | <0.001 |
| F | NV | *nervosa - stenocauda* | PC3 | <0.001 |
| F | NV | *nervosa - zernovi* | PC3 | <0.001 |
| F | NV | *obenbergeri - rectipennis* | PC3 | <0.001 |
| F | NV | *obenbergeri - stenocauda* | PC3 | <0.001 |
| F | NV | *obenbergeri - zernovi* | PC3 | <0.001 |
| F | NV | *rectipennis - stenocauda* | PC3 | <0.001 |
| F | NV | *rectipennis - zernovi* | PC3 | <0.001 |
| F | NV | *stenocauda - zernovi* | PC3 | 1 |
| M | NV | *autumnalis - bicarinata* | PC3 | <0.001 |
| M | NV | *autumnalis - ilkazi* | PC3 | <0.001 |
| M | NV | *autumnalis - karadenizensis* | PC3 | 0.102 |
| M | NV | *autumnalis - nervosa* | PC3 | <0.001 |
| M | NV | *autumnalis - obenbergeri* | PC3 | <0.001 |
| M | NV | *autumnalis - rectipennis* | PC3 | 0.001 |
| M | NV | *autumnalis - stenocauda* | PC3 | <0.001 |
| M | NV | *autumnalis - zernovi* | PC3 | 0.567 |
| M | NV | *bicarinata - ilkazi* | PC3 | <0.001 |
| M | NV | *bicarinata - karadenizensis* | PC3 | <0.001 |
| M | NV | *bicarinata - nervosa* | PC3 | <0.001 |
| M | NV | *bicarinata - obenbergeri* | PC3 | <0.001 |
| M | NV | *bicarinata - rectipennis* | PC3 | 0.284 |
| M | NV | *bicarinata - stenocauda* | PC3 | <0.001 |
| M | NV | *bicarinata - zernovi* | PC3 | <0.001 |
| M | NV | *ilkazi - karadenizensis* | PC3 | <0.001 |
| M | NV | *ilkazi - nervosa* | PC3 | <0.001 |
| M | NV | *ilkazi - obenbergeri* | PC3 | <0.001 |
| M | NV | *ilkazi - rectipennis* | PC3 | <0.001 |
| M | NV | *ilkazi - stenocauda* | PC3 | <0.001 |
| M | NV | *ilkazi - zernovi* | PC3 | <0.001 |
| M | NV | *karadenizensis - nervosa* | PC3 | 0.284 |
| M | NV | *karadenizensis - obenbergeri* | PC3 | <0.001 |
| M | NV | *karadenizensis - rectipennis* | PC3 | <0.001 |
| M | NV | *karadenizensis - stenocauda* | PC3 | <0.001 |
| M | NV | *karadenizensis - zernovi* | PC3 | 0.284 |
| M | NV | *nervosa - obenbergeri* | PC3 | <0.001 |
| M | NV | *nervosa - rectipennis* | PC3 | <0.001 |
| M | NV | *nervosa - stenocauda* | PC3 | 0.143 |
| M | NV | *nervosa - zernovi* | PC3 | 0.004 |
| M | NV | *obenbergeri - rectipennis* | PC3 | <0.001 |
| M | NV | *obenbergeri - stenocauda* | PC3 | <0.001 |
| M | NV | *obenbergeri - zernovi* | PC3 | <0.001 |
| M | NV | *rectipennis - stenocauda* | PC3 | <0.001 |
| M | NV | *rectipennis - zernovi* | PC3 | <0.001 |
| M | NV | *stenocauda - zernovi* | PC3 | <0.001 |
| F | V | *autumnalis - bicarinata* | PC3 | 0.868 |
| F | V | *autumnalis - ilkazi* | PC3 | <0.001 |
| F | V | *autumnalis - karadenizensis* | PC3 | 0.327 |
| F | V | *autumnalis - nervosa* | PC3 | 0.012 |
| F | V | *autumnalis - obenbergeri* | PC3 | <0.001 |
| F | V | *autumnalis - rectipennis* | PC3 | <0.001 |
| F | V | *autumnalis - staneki* | PC3 | <0.001 |
| F | V | *autumnalis - stenocauda* | PC3 | 0.041 |
| F | V | *autumnalis - zernovi* | PC3 | 1 |
| F | V | *bicarinata - ilkazi* | PC3 | <0.001 |
| F | V | *bicarinata - karadenizensis* | PC3 | 0.011 |
| F | V | *bicarinata - nervosa* | PC3 | 0.327 |
| F | V | *bicarinata - obenbergeri* | PC3 | <0.001 |
| F | V | *bicarinata - rectipennis* | PC3 | <0.001 |
| F | V | *bicarinata - staneki* | PC3 | <0.001 |
| F | V | *bicarinata - stenocauda* | PC3 | <0.001 |
| F | V | *bicarinata - zernovi* | PC3 | 0.231 |
| F | V | *ilkazi - karadenizensis* | PC3 | <0.001 |
| F | V | *ilkazi - nervosa* | PC3 | <0.001 |
| F | V | *ilkazi - obenbergeri* | PC3 | <0.001 |
| F | V | *ilkazi - rectipennis* | PC3 | <0.001 |
| F | V | *ilkazi - staneki* | PC3 | <0.001 |
| F | V | *ilkazi - stenocauda* | PC3 | <0.001 |
| F | V | *ilkazi - zernovi* | PC3 | <0.001 |
| F | V | *karadenizensis - nervosa* | PC3 | <0.001 |
| F | V | *karadenizensis - obenbergeri* | PC3 | <0.001 |
| F | V | *karadenizensis - rectipennis* | PC3 | <0.001 |
| F | V | *karadenizensis - staneki* | PC3 | 0.002 |
| F | V | *karadenizensis - stenocauda* | PC3 | 1 |
| F | V | *karadenizensis - zernovi* | PC3 | 0.885 |
| F | V | *nervosa - obenbergeri* | PC3 | <0.001 |
| F | V | *nervosa - rectipennis* | PC3 | <0.001 |
| F | V | *nervosa - staneki* | PC3 | <0.001 |
| F | V | *nervosa - stenocauda* | PC3 | <0.001 |
| F | V | *nervosa - zernovi* | PC3 | <0.001 |
| F | V | *obenbergeri - rectipennis* | PC3 | <0.001 |
| F | V | *obenbergeri - staneki* | PC3 | 1 |
| F | V | *obenbergeri - stenocauda* | PC3 | <0.001 |
| F | V | *obenbergeri - zernovi* | PC3 | <0.001 |
| F | V | *rectipennis - staneki* | PC3 | <0.001 |
| F | V | *rectipennis - stenocauda* | PC3 | <0.001 |
| F | V | *rectipennis - zernovi* | PC3 | <0.001 |
| F | V | *staneki - stenocauda* | PC3 | 0.007 |
| F | V | *staneki - zernovi* | PC3 | <0.001 |
| F | V | *stenocauda - zernovi* | PC3 | 0.327 |
| M | V | *autumnalis - bicarinata* | PC3 | <0.001 |
| M | V | *autumnalis - ilkazi* | PC3 | <0.001 |
| M | V | *autumnalis - karadenizensis* | PC3 | 1 |
| M | V | *autumnalis - nervosa* | PC3 | <0.001 |
| M | V | *autumnalis - obenbergeri* | PC3 | <0.001 |
| M | V | *autumnalis - rectipennis* | PC3 | 0.711 |
| M | V | *autumnalis - staneki* | PC3 | <0.001 |
| M | V | *autumnalis - stenocauda* | PC3 | <0.001 |
| M | V | *autumnalis - zernovi* | PC3 | 1 |
| M | V | *bicarinata - ilkazi* | PC3 | <0.001 |
| M | V | *bicarinata - karadenizensis* | PC3 | <0.001 |
| M | V | *bicarinata - nervosa* | PC3 | <0.001 |
| M | V | *bicarinata - obenbergeri* | PC3 | <0.001 |
| M | V | *bicarinata - rectipennis* | PC3 | <0.001 |
| M | V | *bicarinata - staneki* | PC3 | <0.001 |
| M | V | *bicarinata - stenocauda* | PC3 | <0.001 |
| M | V | *bicarinata - zernovi* | PC3 | <0.001 |
| M | V | *ilkazi - karadenizensis* | PC3 | <0.001 |
| M | V | *ilkazi - nervosa* | PC3 | <0.001 |
| M | V | *ilkazi - obenbergeri* | PC3 | 1 |
| M | V | *ilkazi - rectipennis* | PC3 | <0.001 |
| M | V | *ilkazi - staneki* | PC3 | <0.001 |
| M | V | *ilkazi - stenocauda* | PC3 | <0.001 |
| M | V | *ilkazi - zernovi* | PC3 | <0.001 |
| M | V | *karadenizensis - nervosa* | PC3 | <0.001 |
| M | V | *karadenizensis - obenbergeri* | PC3 | <0.001 |
| M | V | *karadenizensis - rectipennis* | PC3 | 0.064 |
| M | V | *karadenizensis - staneki* | PC3 | <0.001 |
| M | V | *karadenizensis - stenocauda* | PC3 | 0.002 |
| M | V | *karadenizensis - zernovi* | PC3 | 0.715 |
| M | V | *nervosa - obenbergeri* | PC3 | <0.001 |
| M | V | *nervosa - rectipennis* | PC3 | <0.001 |
| M | V | *nervosa - staneki* | PC3 | 0.015 |
| M | V | *nervosa - stenocauda* | PC3 | 0.715 |
| M | V | *nervosa - zernovi* | PC3 | <0.001 |
| M | V | *obenbergeri - rectipennis* | PC3 | <0.001 |
| M | V | *obenbergeri - staneki* | PC3 | <0.001 |
| M | V | *obenbergeri - stenocauda* | PC3 | <0.001 |
| M | V | *obenbergeri - zernovi* | PC3 | <0.001 |
| M | V | *rectipennis - staneki* | PC3 | <0.001 |
| M | V | *rectipennis - stenocauda* | PC3 | <0.001 |
| M | V | *rectipennis - zernovi* | PC3 | 1 |
| M | V | *staneki - stenocauda* | PC3 | <0.001 |
| M | V | *staneki - zernovi* | PC3 | <0.001 |
| M | V | *stenocauda - zernovi* | PC3 | <0.001 |
| F | NV | *autumnalis - bicarinata* | PC4 | 0.003 |
| F | NV | *autumnalis - ilkazi* | PC4 | 0.019 |
| F | NV | *autumnalis - karadenizensis* | PC4 | 0.136 |
| F | NV | *autumnalis - nervosa* | PC4 | <0.001 |
| F | NV | *autumnalis - obenbergeri* | PC4 | <0.001 |
| F | NV | *autumnalis - rectipennis* | PC4 | <0.001 |
| F | NV | *autumnalis - stenocauda* | PC4 | <0.001 |
| F | NV | *autumnalis - zernovi* | PC4 | 0.858 |
| F | NV | *bicarinata - ilkazi* | PC4 | <0.001 |
| F | NV | *bicarinata - karadenizensis* | PC4 | 0.526 |
| F | NV | *bicarinata - nervosa* | PC4 | <0.001 |
| F | NV | *bicarinata - obenbergeri* | PC4 | <0.001 |
| F | NV | *bicarinata - rectipennis* | PC4 | <0.001 |
| F | NV | *bicarinata - stenocauda* | PC4 | <0.001 |
| F | NV | *bicarinata - zernovi* | PC4 | 0.023 |
| F | NV | *ilkazi - karadenizensis* | PC4 | <0.001 |
| F | NV | *ilkazi - nervosa* | PC4 | <0.001 |
| F | NV | *ilkazi - obenbergeri* | PC4 | <0.001 |
| F | NV | *ilkazi - rectipennis* | PC4 | <0.001 |
| F | NV | *ilkazi - stenocauda* | PC4 | 0.115 |
| F | NV | *ilkazi - zernovi* | PC4 | <0.001 |
| F | NV | *karadenizensis - nervosa* | PC4 | <0.001 |
| F | NV | *karadenizensis - obenbergeri* | PC4 | <0.001 |
| F | NV | *karadenizensis - rectipennis* | PC4 | <0.001 |
| F | NV | *karadenizensis - stenocauda* | PC4 | <0.001 |
| F | NV | *karadenizensis - zernovi* | PC4 | 0.477 |
| F | NV | *nervosa - obenbergeri* | PC4 | 0.858 |
| F | NV | *nervosa - rectipennis* | PC4 | 0.136 |
| F | NV | *nervosa - stenocauda* | PC4 | <0.001 |
| F | NV | *nervosa - zernovi* | PC4 | <0.001 |
| F | NV | *obenbergeri - rectipennis* | PC4 | 0.504 |
| F | NV | *obenbergeri - stenocauda* | PC4 | <0.001 |
| F | NV | *obenbergeri - zernovi* | PC4 | <0.001 |
| F | NV | *rectipennis - stenocauda* | PC4 | 0.002 |
| F | NV | *rectipennis - zernovi* | PC4 | <0.001 |
| F | NV | *stenocauda - zernovi* | PC4 | <0.001 |
| M | NV | *autumnalis - bicarinata* | PC4 | <0.001 |
| M | NV | *autumnalis - ilkazi* | PC4 | <0.001 |
| M | NV | *autumnalis - karadenizensis* | PC4 | 0.88 |
| M | NV | *autumnalis - nervosa* | PC4 | 1 |
| M | NV | *autumnalis - obenbergeri* | PC4 | <0.001 |
| M | NV | *autumnalis - rectipennis* | PC4 | <0.001 |
| M | NV | *autumnalis - stenocauda* | PC4 | 0.093 |
| M | NV | *autumnalis - zernovi* | PC4 | 1 |
| M | NV | *bicarinata - ilkazi* | PC4 | 0.535 |
| M | NV | *bicarinata - karadenizensis* | PC4 | <0.001 |
| M | NV | *bicarinata - nervosa* | PC4 | <0.001 |
| M | NV | *bicarinata - obenbergeri* | PC4 | <0.001 |
| M | NV | *bicarinata - rectipennis* | PC4 | <0.001 |
| M | NV | *bicarinata - stenocauda* | PC4 | <0.001 |
| M | NV | *bicarinata - zernovi* | PC4 | <0.001 |
| M | NV | *ilkazi - karadenizensis* | PC4 | <0.001 |
| M | NV | *ilkazi - nervosa* | PC4 | <0.001 |
| M | NV | *ilkazi - obenbergeri* | PC4 | 0.843 |
| M | NV | *ilkazi - rectipennis* | PC4 | 0.484 |
| M | NV | *ilkazi - stenocauda* | PC4 | 0.002 |
| M | NV | *ilkazi - zernovi* | PC4 | <0.001 |
| M | NV | *karadenizensis - nervosa* | PC4 | 1 |
| M | NV | *karadenizensis - obenbergeri* | PC4 | 0.004 |
| M | NV | *karadenizensis - rectipennis* | PC4 | 0.015 |
| M | NV | *karadenizensis - stenocauda* | PC4 | 1 |
| M | NV | *karadenizensis - zernovi* | PC4 | 1 |
| M | NV | *nervosa - obenbergeri* | PC4 | 0.001 |
| M | NV | *nervosa - rectipennis* | PC4 | 0.004 |
| M | NV | *nervosa - stenocauda* | PC4 | 0.867 |
| M | NV | *nervosa - zernovi* | PC4 | 1 |
| M | NV | *obenbergeri - rectipennis* | PC4 | 1 |
| M | NV | *obenbergeri - stenocauda* | PC4 | 0.264 |
| M | NV | *obenbergeri - zernovi* | PC4 | 0.003 |
| M | NV | *rectipennis - stenocauda* | PC4 | 0.551 |
| M | NV | *rectipennis - zernovi* | PC4 | 0.012 |
| M | NV | *stenocauda - zernovi* | PC4 | 1 |
| F | V | *autumnalis - bicarinata* | PC4 | <0.001 |
| F | V | *autumnalis - ilkazi* | PC4 | 1 |
| F | V | *autumnalis - karadenizensis* | PC4 | <0.001 |
| F | V | *autumnalis - nervosa* | PC4 | <0.001 |
| F | V | *autumnalis - obenbergeri* | PC4 | <0.001 |
| F | V | *autumnalis - rectipennis* | PC4 | <0.001 |
| F | V | *autumnalis - staneki* | PC4 | <0.001 |
| F | V | *autumnalis - stenocauda* | PC4 | 0.024 |
| F | V | *autumnalis - zernovi* | PC4 | 0.002 |
| F | V | *bicarinata - ilkazi* | PC4 | 0.001 |
| F | V | *bicarinata - karadenizensis* | PC4 | 0.188 |
| F | V | *bicarinata - nervosa* | PC4 | <0.001 |
| F | V | *bicarinata - obenbergeri* | PC4 | <0.001 |
| F | V | *bicarinata - rectipennis* | PC4 | <0.001 |
| F | V | *bicarinata - staneki* | PC4 | <0.001 |
| F | V | *bicarinata - stenocauda* | PC4 | 0.646 |
| F | V | *bicarinata - zernovi* | PC4 | 1 |
| F | V | *ilkazi - karadenizensis* | PC4 | <0.001 |
| F | V | *ilkazi - nervosa* | PC4 | <0.001 |
| F | V | *ilkazi - obenbergeri* | PC4 | <0.001 |
| F | V | *ilkazi - rectipennis* | PC4 | <0.001 |
| F | V | *ilkazi - staneki* | PC4 | <0.001 |
| F | V | *ilkazi - stenocauda* | PC4 | 0.205 |
| F | V | *ilkazi - zernovi* | PC4 | 0.033 |
| F | V | *karadenizensis - nervosa* | PC4 | <0.001 |
| F | V | *karadenizensis - obenbergeri* | PC4 | <0.001 |
| F | V | *karadenizensis - rectipennis* | PC4 | <0.001 |
| F | V | *karadenizensis - staneki* | PC4 | <0.001 |
| F | V | *karadenizensis - stenocauda* | PC4 | 0.001 |
| F | V | *karadenizensis - zernovi* | PC4 | 0.014 |
| F | V | *nervosa - obenbergeri* | PC4 | 0.033 |
| F | V | *nervosa - rectipennis* | PC4 | 0.002 |
| F | V | *nervosa - staneki* | PC4 | 1 |
| F | V | *nervosa - stenocauda* | PC4 | <0.001 |
| F | V | *nervosa - zernovi* | PC4 | <0.001 |
| F | V | *obenbergeri - rectipennis* | PC4 | 1 |
| F | V | *obenbergeri - staneki* | PC4 | 1 |
| F | V | *obenbergeri - stenocauda* | PC4 | <0.001 |
| F | V | *obenbergeri - zernovi* | PC4 | <0.001 |
| F | V | *rectipennis - staneki* | PC4 | 1 |
| F | V | *rectipennis - stenocauda* | PC4 | <0.001 |
| F | V | *rectipennis - zernovi* | PC4 | <0.001 |
| F | V | *staneki - stenocauda* | PC4 | <0.001 |
| F | V | *staneki - zernovi* | PC4 | <0.001 |
| F | V | *stenocauda - zernovi* | PC4 | 1 |
| M | V | *autumnalis - bicarinata* | PC4 | <0.001 |
| M | V | *autumnalis - ilkazi* | PC4 | <0.001 |
| M | V | *autumnalis - karadenizensis* | PC4 | 1 |
| M | V | *autumnalis - nervosa* | PC4 | 1 |
| M | V | *autumnalis - obenbergeri* | PC4 | <0.001 |
| M | V | *autumnalis - rectipennis* | PC4 | 1 |
| M | V | *autumnalis - staneki* | PC4 | 1 |
| M | V | *autumnalis - stenocauda* | PC4 | 0.059 |
| M | V | *autumnalis - zernovi* | PC4 | 0.847 |
| M | V | *bicarinata - ilkazi* | PC4 | 0.526 |
| M | V | *bicarinata - karadenizensis* | PC4 | <0.001 |
| M | V | *bicarinata - nervosa* | PC4 | <0.001 |
| M | V | *bicarinata - obenbergeri* | PC4 | 1 |
| M | V | *bicarinata - rectipennis* | PC4 | <0.001 |
| M | V | *bicarinata - staneki* | PC4 | <0.001 |
| M | V | *bicarinata - stenocauda* | PC4 | 0.015 |
| M | V | *bicarinata - zernovi* | PC4 | <0.001 |
| M | V | *ilkazi - karadenizensis* | PC4 | <0.001 |
| M | V | *ilkazi - nervosa* | PC4 | 0.001 |
| M | V | *ilkazi - obenbergeri* | PC4 | 1 |
| M | V | *ilkazi - rectipennis* | PC4 | <0.001 |
| M | V | *ilkazi - staneki* | PC4 | 0.001 |
| M | V | *ilkazi - stenocauda* | PC4 | 1 |
| M | V | *ilkazi - zernovi* | PC4 | 0.281 |
| M | V | *karadenizensis - nervosa* | PC4 | 1 |
| M | V | *karadenizensis - obenbergeri* | PC4 | <0.001 |
| M | V | *karadenizensis - rectipennis* | PC4 | 1 |
| M | V | *karadenizensis - staneki* | PC4 | 1 |
| M | V | *karadenizensis - stenocauda* | PC4 | 0.001 |
| M | V | *karadenizensis - zernovi* | PC4 | 0.051 |
| M | V | *nervosa - obenbergeri* | PC4 | <0.001 |
| M | V | *nervosa - rectipennis* | PC4 | 1 |
| M | V | *nervosa - staneki* | PC4 | 1 |
| M | V | *nervosa - stenocauda* | PC4 | 0.107 |
| M | V | *nervosa - zernovi* | PC4 | 1 |
| M | V | *obenbergeri - rectipennis* | PC4 | <0.001 |
| M | V | *obenbergeri - staneki* | PC4 | <0.001 |
| M | V | *obenbergeri - stenocauda* | PC4 | 0.312 |
| M | V | *obenbergeri - zernovi* | PC4 | 0.012 |
| M | V | *rectipennis - staneki* | PC4 | 1 |
| M | V | *rectipennis - stenocauda* | PC4 | 0.02 |
| M | V | *rectipennis - zernovi* | PC4 | 0.406 |
| M | V | *staneki - stenocauda* | PC4 | 0.019 |
| M | V | *staneki - zernovi* | PC4 | 0.126 |
| M | V | *stenocauda - zernovi* | PC4 | 1 |
| F | NV | *autumnalis - bicarinata* | PC5 | 1 |
| F | NV | *autumnalis - ilkazi* | PC5 | 0.008 |
| F | NV | *autumnalis - karadenizensis* | PC5 | <0.001 |
| F | NV | *autumnalis - nervosa* | PC5 | <0.001 |
| F | NV | *autumnalis - obenbergeri* | PC5 | <0.001 |
| F | NV | *autumnalis - rectipennis* | PC5 | <0.001 |
| F | NV | *autumnalis - stenocauda* | PC5 | 0.004 |
| F | NV | *autumnalis - zernovi* | PC5 | <0.001 |
| F | NV | *bicarinata - ilkazi* | PC5 | 0.001 |
| F | NV | *bicarinata - karadenizensis* | PC5 | 0.001 |
| F | NV | *bicarinata - nervosa* | PC5 | <0.001 |
| F | NV | *bicarinata - obenbergeri* | PC5 | <0.001 |
| F | NV | *bicarinata - rectipennis* | PC5 | <0.001 |
| F | NV | *bicarinata - stenocauda* | PC5 | 0.008 |
| F | NV | *bicarinata - zernovi* | PC5 | <0.001 |
| F | NV | *ilkazi - karadenizensis* | PC5 | <0.001 |
| F | NV | *ilkazi - nervosa* | PC5 | <0.001 |
| F | NV | *ilkazi - obenbergeri* | PC5 | <0.001 |
| F | NV | *ilkazi - rectipennis* | PC5 | <0.001 |
| F | NV | *ilkazi - stenocauda* | PC5 | <0.001 |
| F | NV | *ilkazi - zernovi* | PC5 | <0.001 |
| F | NV | *karadenizensis - nervosa* | PC5 | <0.001 |
| F | NV | *karadenizensis - obenbergeri* | PC5 | <0.001 |
| F | NV | *karadenizensis - rectipennis* | PC5 | 1 |
| F | NV | *karadenizensis - stenocauda* | PC5 | 1 |
| F | NV | *karadenizensis - zernovi* | PC5 | 0.063 |
| F | NV | *nervosa - obenbergeri* | PC5 | <0.001 |
| F | NV | *nervosa - rectipennis* | PC5 | <0.001 |
| F | NV | *nervosa - stenocauda* | PC5 | <0.001 |
| F | NV | *nervosa - zernovi* | PC5 | <0.001 |
| F | NV | *obenbergeri - rectipennis* | PC5 | <0.001 |
| F | NV | *obenbergeri - stenocauda* | PC5 | <0.001 |
| F | NV | *obenbergeri - zernovi* | PC5 | 0.04 |
| F | NV | *rectipennis - stenocauda* | PC5 | 1 |
| F | NV | *rectipennis - zernovi* | PC5 | 0.438 |
| F | NV | *stenocauda - zernovi* | PC5 | 0.04 |
| M | NV | *autumnalis - bicarinata* | PC5 | 0.92 |
| M | NV | *autumnalis - ilkazi* | PC5 | 1 |
| M | NV | *autumnalis - karadenizensis* | PC5 | <0.001 |
| M | NV | *autumnalis - nervosa* | PC5 | 0.158 |
| M | NV | *autumnalis - obenbergeri* | PC5 | <0.001 |
| M | NV | *autumnalis - rectipennis* | PC5 | <0.001 |
| M | NV | *autumnalis - stenocauda* | PC5 | <0.001 |
| M | NV | *autumnalis - zernovi* | PC5 | 0.158 |
| M | NV | *bicarinata - ilkazi* | PC5 | 0.24 |
| M | NV | *bicarinata - karadenizensis* | PC5 | <0.001 |
| M | NV | *bicarinata - nervosa* | PC5 | 1 |
| M | NV | *bicarinata - obenbergeri* | PC5 | <0.001 |
| M | NV | *bicarinata - rectipennis* | PC5 | <0.001 |
| M | NV | *bicarinata - stenocauda* | PC5 | <0.001 |
| M | NV | *bicarinata - zernovi* | PC5 | 0.001 |
| M | NV | *ilkazi - karadenizensis* | PC5 | 0.171 |
| M | NV | *ilkazi - nervosa* | PC5 | 0.029 |
| M | NV | *ilkazi - obenbergeri* | PC5 | <0.001 |
| M | NV | *ilkazi - rectipennis* | PC5 | 0.142 |
| M | NV | *ilkazi - stenocauda* | PC5 | <0.001 |
| M | NV | *ilkazi - zernovi* | PC5 | 1 |
| M | NV | *karadenizensis - nervosa* | PC5 | <0.001 |
| M | NV | *karadenizensis - obenbergeri* | PC5 | <0.001 |
| M | NV | *karadenizensis - rectipennis* | PC5 | 1 |
| M | NV | *karadenizensis - stenocauda* | PC5 | <0.001 |
| M | NV | *karadenizensis - zernovi* | PC5 | 0.808 |
| M | NV | *nervosa - obenbergeri* | PC5 | <0.001 |
| M | NV | *nervosa - rectipennis* | PC5 | <0.001 |
| M | NV | *nervosa - stenocauda* | PC5 | <0.001 |
| M | NV | *nervosa - zernovi* | PC5 | <0.001 |
| M | NV | *obenbergeri - rectipennis* | PC5 | <0.001 |
| M | NV | *obenbergeri - stenocauda* | PC5 | 1 |
| M | NV | *obenbergeri - zernovi* | PC5 | <0.001 |
| M | NV | *rectipennis - stenocauda* | PC5 | <0.001 |
| M | NV | *rectipennis - zernovi* | PC5 | 0.587 |
| M | NV | *stenocauda - zernovi* | PC5 | <0.001 |
| F | V | *autumnalis - bicarinata* | PC5 | <0.001 |
| F | V | *autumnalis - ilkazi* | PC5 | <0.001 |
| F | V | *autumnalis - karadenizensis* | PC5 | <0.001 |
| F | V | *autumnalis - nervosa* | PC5 | 0.364 |
| F | V | *autumnalis - obenbergeri* | PC5 | <0.001 |
| F | V | *autumnalis - rectipennis* | PC5 | <0.001 |
| F | V | *autumnalis - staneki* | PC5 | <0.001 |
| F | V | *autumnalis - stenocauda* | PC5 | <0.001 |
| F | V | *autumnalis - zernovi* | PC5 | <0.001 |
| F | V | *bicarinata - ilkazi* | PC5 | <0.001 |
| F | V | *bicarinata - karadenizensis* | PC5 | 1 |
| F | V | *bicarinata - nervosa* | PC5 | <0.001 |
| F | V | *bicarinata - obenbergeri* | PC5 | <0.001 |
| F | V | *bicarinata - rectipennis* | PC5 | <0.001 |
| F | V | *bicarinata - staneki* | PC5 | 0.066 |
| F | V | *bicarinata - stenocauda* | PC5 | 1 |
| F | V | *bicarinata - zernovi* | PC5 | <0.001 |
| F | V | *ilkazi - karadenizensis* | PC5 | <0.001 |
| F | V | *ilkazi - nervosa* | PC5 | <0.001 |
| F | V | *ilkazi - obenbergeri* | PC5 | <0.001 |
| F | V | *ilkazi - rectipennis* | PC5 | <0.001 |
| F | V | *ilkazi - staneki* | PC5 | <0.001 |
| F | V | *ilkazi - stenocauda* | PC5 | <0.001 |
| F | V | *ilkazi - zernovi* | PC5 | <0.001 |
| F | V | *karadenizensis - nervosa* | PC5 | <0.001 |
| F | V | *karadenizensis - obenbergeri* | PC5 | <0.001 |
| F | V | *karadenizensis - rectipennis* | PC5 | <0.001 |
| F | V | *karadenizensis - staneki* | PC5 | 0.034 |
| F | V | *karadenizensis - stenocauda* | PC5 | 1 |
| F | V | *karadenizensis - zernovi* | PC5 | <0.001 |
| F | V | *nervosa - obenbergeri* | PC5 | <0.001 |
| F | V | *nervosa - rectipennis* | PC5 | <0.001 |
| F | V | *nervosa - staneki* | PC5 | <0.001 |
| F | V | *nervosa - stenocauda* | PC5 | <0.001 |
| F | V | *nervosa - zernovi* | PC5 | <0.001 |
| F | V | *obenbergeri - rectipennis* | PC5 | 0.051 |
| F | V | *obenbergeri - staneki* | PC5 | 0.314 |
| F | V | *obenbergeri - stenocauda* | PC5 | <0.001 |
| F | V | *obenbergeri - zernovi* | PC5 | 0.364 |
| F | V | *rectipennis - staneki* | PC5 | 1 |
| F | V | *rectipennis - stenocauda* | PC5 | <0.001 |
| F | V | *rectipennis - zernovi* | PC5 | <0.001 |
| F | V | *staneki - stenocauda* | PC5 | 0.054 |
| F | V | *staneki - zernovi* | PC5 | 0.025 |
| F | V | *stenocauda - zernovi* | PC5 | <0.001 |
| M | V | *autumnalis - bicarinata* | PC5 | 0.001 |
| M | V | *autumnalis - ilkazi* | PC5 | 0.017 |
| M | V | *autumnalis - karadenizensis* | PC5 | 1 |
| M | V | *autumnalis - nervosa* | PC5 | <0.001 |
| M | V | *autumnalis - obenbergeri* | PC5 | <0.001 |
| M | V | *autumnalis - rectipennis* | PC5 | <0.001 |
| M | V | *autumnalis - staneki* | PC5 | 0.435 |
| M | V | *autumnalis - stenocauda* | PC5 | <0.001 |
| M | V | *autumnalis - zernovi* | PC5 | 0.189 |
| M | V | *bicarinata - ilkazi* | PC5 | 1 |
| M | V | *bicarinata - karadenizensis* | PC5 | <0.001 |
| M | V | *bicarinata - nervosa* | PC5 | 0.484 |
| M | V | *bicarinata - obenbergeri* | PC5 | <0.001 |
| M | V | *bicarinata - rectipennis* | PC5 | 0.07 |
| M | V | *bicarinata - staneki* | PC5 | <0.001 |
| M | V | *bicarinata - stenocauda* | PC5 | <0.001 |
| M | V | *bicarinata - zernovi* | PC5 | <0.001 |
| M | V | *ilkazi - karadenizensis* | PC5 | 0.003 |
| M | V | *ilkazi - nervosa* | PC5 | 0.091 |
| M | V | *ilkazi - obenbergeri* | PC5 | <0.001 |
| M | V | *ilkazi - rectipennis* | PC5 | 0.006 |
| M | V | *ilkazi - staneki* | PC5 | 0.001 |
| M | V | *ilkazi - stenocauda* | PC5 | <0.001 |
| M | V | *ilkazi - zernovi* | PC5 | <0.001 |
| M | V | *karadenizensis - nervosa* | PC5 | <0.001 |
| M | V | *karadenizensis - obenbergeri* | PC5 | 0.001 |
| M | V | *karadenizensis - rectipennis* | PC5 | <0.001 |
| M | V | *karadenizensis - staneki* | PC5 | 0.578 |
| M | V | *karadenizensis - stenocauda* | PC5 | 0.003 |
| M | V | *karadenizensis - zernovi* | PC5 | 0.489 |
| M | V | *nervosa - obenbergeri* | PC5 | <0.001 |
| M | V | *nervosa - rectipennis* | PC5 | 1 |
| M | V | *nervosa - staneki* | PC5 | <0.001 |
| M | V | *nervosa - stenocauda* | PC5 | <0.001 |
| M | V | *nervosa - zernovi* | PC5 | <0.001 |
| M | V | *obenbergeri - rectipennis* | PC5 | <0.001 |
| M | V | *obenbergeri - staneki* | PC5 | 1 |
| M | V | *obenbergeri - stenocauda* | PC5 | 1 |
| M | V | *obenbergeri - zernovi* | PC5 | 0.48 |
| M | V | *rectipennis - staneki* | PC5 | <0.001 |
| M | V | *rectipennis - stenocauda* | PC5 | <0.001 |
| M | V | *rectipennis - zernovi* | PC5 | <0.001 |
| M | V | *staneki - stenocauda* | PC5 | 1 |
| M | V | *staneki - zernovi* | PC5 | 1 |
| M | V | *stenocauda - zernovi* | PC5 | 0.578 |
