# Supplementary material for: Cuticular hydrocarbon profiles in plump bush crickets vary according to species, sex and mating status
Source: Sci Rep. 2025 Sep 26;15:33233. doi: 10.1038/s41598-025-17544-7 (PMC12475074; doi:10.1038/s41598-025-17544-7)
Supplement: Supplementary file 6 — Supplementary Material 6 [file 41598_2025_17544_MOESM6_ESM.docx]

**Supp. Table 3.** Pairwise comparisons of multivariate dispersion among species based on CHC profiles. We performed a multivariate homogeneity of dispersion analysis (betadisper) using Bray–Curtis distances calculated from CHC profiles (log-transformed peak areas). Pairwise species comparisons were evaluated with Tukey’s HSD test to identify significant differences in dispersion around group centroids.

| **Species Comparisons** | **Difference in Dispersion** | **Lower CI** | **Upper CI** | **Adjusted p-value** |
| --- | --- | --- | --- | --- |
| *bicarinata-autumnalis* | 0.059 | 0.025 | 0.093 | <0.001 |
| *ilkazi-autumnalis* | 0.007 | -0.029 | 0.044 | 1.000 |
| *karadenizensis-autumnalis* | 0.116 | 0.081 | 0.150 | <0.001 |
| *nervosa-autumnalis* | -0.088 | -0.123 | -0.053 | <0.001 |
| *obenbergeri-autumnalis* | -0.041 | -0.076 | -0.006 | 0.007 |
| *rectipennis-autumnalis* | -0.057 | -0.092 | -0.022 | <0.001 |
| *staneki-autumnalis* | -0.127 | -0.206 | -0.049 | <0.001 |
| *stenocauda-autumnalis* | 0.003 | -0.032 | 0.038 | 1.000 |
| *zernovi-autumnalis* | 0.024 | -0.010 | 0.058 | 0.404 |
| *ilkazi-bicarinata* | -0.052 | -0.088 | -0.016 | <0.001 |
| *karadenizensis-bicarinata* | 0.056 | 0.023 | 0.090 | <0.001 |
| *nervosa-bicarinata* | -0.147 | -0.182 | -0.113 | <0.001 |
| *obenbergeri-bicarinata* | -0.100 | -0.135 | -0.066 | <0.001 |
| *rectipennis-bicarinata* | -0.116 | -0.150 | -0.082 | <0.001 |
| *staneki-bicarinata* | -0.186 | -0.265 | -0.108 | <0.001 |
| *stenocauda-bicarinata* | -0.056 | -0.091 | -0.022 | <0.001 |
| *zernovi-bicarinata* | -0.035 | -0.068 | -0.001 | 0.034 |
| *karadenizensis-ilkazi* | 0.108 | 0.072 | 0.144 | <0.001 |
| *nervosa-ilkazi* | -0.096 | -0.133 | -0.059 | <0.001 |
| *obenbergeri-ilkazi* | -0.049 | -0.085 | -0.012 | 0.001 |
| *rectipennis-ilkazi* | -0.065 | -0.101 | -0.028 | <0.001 |
| *staneki-ilkazi* | -0.135 | -0.214 | -0.055 | <0.001 |
| *stenocauda-ilkazi* | -0.005 | -0.042 | 0.032 | 1.000 |
| *zernovi-ilkazi* | 0.017 | -0.019 | 0.053 | 0.892 |
| *nervosa-karadenizensis* | -0.204 | -0.239 | -0.169 | <0.001 |
| *obenbergeri-karadenizensis* | -0.157 | -0.191 | -0.122 | <0.001 |
| *rectipennis-karadenizensis* | -0.173 | -0.207 | -0.138 | <0.001 |
| *staneki-karadenizensis* | -0.243 | -0.321 | -0.164 | <0.001 |
| *stenocauda-karadenizensis* | -0.113 | -0.148 | -0.078 | <0.001 |
| *zernovi-karadenizensis* | -0.091 | -0.125 | -0.057 | <0.001 |
| *obenbergeri-nervosa* | 0.047 | 0.012 | 0.083 | 0.001 |
| *rectipennis-nervosa* | 0.031 | -0.004 | 0.067 | 0.141 |
| *staneki-nervosa* | -0.039 | -0.118 | 0.040 | 0.861 |
| *stenocauda-nervosa* | 0.091 | 0.055 | 0.126 | <0.001 |
| *zernovi-nervosa* | 0.113 | 0.078 | 0.147 | <0.001 |
| *rectipennis-obenbergeri* | -0.016 | -0.051 | 0.019 | 0.915 |
| *staneki-obenbergeri* | -0.086 | -0.165 | -0.008 | 0.019 |
| *stenocauda-obenbergeri* | 0.044 | 0.009 | 0.079 | 0.003 |
| *zernovi-obenbergeri* | 0.066 | 0.031 | 0.100 | <0.001 |
| *staneki-rectipennis* | -0.070 | -0.149 | 0.008 | 0.127 |
| *stenocauda-rectipennis* | 0.060 | 0.025 | 0.095 | <0.001 |
| *zernovi-rectipennis* | 0.082 | 0.047 | 0.116 | <0.001 |
| *stenocauda-staneki* | 0.130 | 0.051 | 0.208 | <0.001 |
| *zernovi-staneki* | 0.152 | 0.074 | 0.230 | <0.001 |
| *zernovi-stenocauda* | 0.022 | -0.013 | 0.056 | 0.593 |
