# Supplementary material for: Cuticular hydrocarbon profiles in plump bush crickets vary according to species, sex and mating status
Source: Sci Rep. 2025 Sep 26;15:33233. doi: 10.1038/s41598-025-17544-7 (PMC12475074; doi:10.1038/s41598-025-17544-7)
Supplement: Supplementary file 7 — Supplementary Material 7 [file 41598_2025_17544_MOESM7_ESM.docx]

**Supplementary Tables**

Supp. Table 1. Mean relative abundance (%) of each CHC class in *Isophya* species, grouped by species, species group, sex, and mating status. Values represent the average percentage contribution of each CHC class to the total cuticular hydrocarbon (CHC) profile per individual. Standard deviations and sample sizes are also provided.Supp. Table 2. Distribution of CHC Classes by species, species group, sex, and mating status.

Supp. Table 2. Results of pairwise species comparisons within each combination of sex and mating status across PC1 to PC5. Pairwise comparisons were performed using estimated marginal means (*emmeans*) based on ANOVA models including species, sex, mating status, and all interactions. P-values were adjusted using the *Holm* method to control for family-wise error rate.

Supp. Table 3. Pairwise comparisons of multivariate dispersion among species based on CHC profiles. We performed a multivariate homogeneity of dispersion analysis (*betadisper*) using Bray–Curtis distances calculated from CHC profiles (log-transformed peak areas). Pairwise species comparisons were evaluated with Tukey’s HSD test to identify significant differences in dispersion around group centroids.

**Supplementary Figures**

Supp. Fig. 1. Photographic collage of representative individuals from selected *Isophya* species used in this study. Images illustrate interspecific morphological diversity and sexual dimorphism among members of the *zernovi, rectipennis*, and *staneki* species groups. Not all species included in the CHC analyses are shown (Photo credit: H. Sevgili).

Supp. Fig. 2. Representative GC-MS chromatograms illustrating cuticular hydrocarbon (CHC) peak profiles of male (blue) and female (red) individuals from three *Isophya* species—*I. zernovi* (*zernovi* group), *I. rectipennis* (*rectipennis* group), and *I. staneki* (*staneki* group).

Supp. Fig. 3. Principal Component Analysis (PCA) of CHC profiles in *Isophya* individuals by sex and mating status. A. Virgins, B. Nonvirgins. The plot shows variation in CHC composition across sexes (male, female) and reproductive status (virgin [V], nonvirgin [NV]). Individuals are grouped by sex × mating status combinations, and ellipses represent 95% confidence intervals.
